# A quantitative environmental impact assessment of Australian ultra-processed beverages and impact reduction scenarios

**DOI:** 10.1017/S1368980025000187

**Published:** 2025-02-04

**Authors:** Kim Anastasiou, Michalis Hadjikakou, Ozge Geyik, Gilly A Hendrie, Phillip Baker, Richard Pinter, Mark Lawrence

**Affiliations:** 1 School of Exercise and Nutrition Sciences, Deakin University, Burwood, VIC, Australia; 2 School of Life and Environmental Sciences, Deakin University, Geelong, VIC, Australia; 3 Health and Biosecurity, Commonwealth Scientific and Industrial Research Organisation (CSIRO), Adelaide, SA, Australia; 4 Institute for Physical Activity and Nutrition, Deakin University, Burwood, VIC, Australia; 5 School of Science and Technology, University of New England, Armidale, NSW, Australia

**Keywords:** Ultra-processed beverages, Ultra-processed foods, Environmental impacts, Sustainable food systems

## Abstract

**Objective:**

Ultra-processed beverages (UPB) have known adverse impacts on health, but their impact on the environment is not well understood across different environmental indicators. This study aimed to quantify the environmental impacts of water-based UPB and bottled waters sold in Australia and assess the impacts of various scenarios which may reduce such impacts in the future.

**Design:**

This study presents a quantitative environmental impact assessment of a major sub-category of UPB (water-based UPB, including soft drinks, energy drinks, cordials and fruit drinks) and non-UPB (bottled waters) in Australia. Alternative mitigation scenarios based on existing health and environmental targets were also modelled using sales projections for 2027. Sales data from Euromonitor International were matched with environmental impact data from peer-reviewed lifecycle assessment databases. Environmental impact indicators included greenhouse gas emissions, land use, eutrophication potential, acidification potential, water scarcity and plastic use.

**Setting:**

The Australian beverage supply in 2022 and projected sales for 2027.

**Participants:**

N/A

**Results:**

Environmental impacts of UPB were higher than bottled waters. UPB accounted for 81–99 % of total environmental impacts, partly driven by the volume of sales. Reformulation, reducing UPB consumption and increasing recycling all led to meaningful reductions in environmental impacts but with diverse effects across different environmental indicators. The largest reductions occurred when policy scenarios were combined to represent a suite of policy actions which aimed to meet health and environmental targets (30–82 % environmental savings).

**Conclusions:**

The results indicate that implementing a suite of policies which act to target multiple drivers of environmental harm are likely to lead to the most environmental benefits.

Urgent changes to our global food system are needed to address environmental and health crises such as biodiversity loss, climate change, pollution, malnutrition and diet-related non-communicable diseases^(1–3)^. The Australian agrifood system emits 174 900 kilotons of CO_2_-eq per year (∼1 % of global food system emissions) and accounts for 55 % of total land use in the country^(4)^. In 2018, poor diets were responsible for 5·4 % of the Australian burden of disease, ranked third-highest in preventable factors leading to ill health^(5)^. Finding solutions that have co-benefits for human and planetary health could help address these intersecting challenges.

The need to improve production and reduce consumption of animal-sourced foods and avoid food loss and waste is well established^(1–3)^. A proposed complementary strategy to improve food systems is to reduce the production and consumption of ultra-processed foods (UPF)^(6–8)^. UPF are defined by the NOVA classification as ‘*formulations of ingredients, mostly of exclusive industrial use, that result from a series of industrial processes’*^(9)^. High consumption of UPF is associated with poorer health outcomes, such as type-2 diabetes, cardiovascular diseases, common mental disorder and all-cause mortality^(10)^. Recent evidence suggests that environmental impacts driven by biological, social and commercial drivers of UPF production and consumption occur across the supply chain^(7)^ and are substantial^(6)^.

Ultra-processed beverages (UPB), such as soft drinks, energy drinks, sports drinks, cordials, flavoured waters and fruit drinks, have received significant attention for their health impacts^(11,12)^. In Australia in 2011–2012, UPF accounted for 74 % of dietary energy from free sugars, and much of this sugar stemmed from sugar-sweetened beverages^(13)^. Beverages may impact over-consumption to a greater extent than foods, as beverages do not have the same satiation effects as foods and they do not replace energy from solid foods or result in compensation in subsequent meal consumption^(14)^. Furthermore, assuming that energy and hydration needs are met, UPB provide little nutritional benefits and thus are often superfluous to dietary requirements.

Both sugar-sweetened and non-sugar-sweetened UPB have been the focus of research on health impacts^(11,12)^ and public health policies^(11,15)^. Most dietary guidelines recommend the consumption of water as a beverage of choice, and many recommend avoiding sugary drinks^(15)^, which are commonly UPB. Despite this, the consumption of UPB in Australia rose by 16·3 g per capita per day between 2018 and 2021^(16)^. Previous studies analysing the environmental impacts of beverages have found that tap water has lower impacts compared with bottled water^(17–19)^, both of which have lower impacts than soft drinks^(17)^. Thus, reducing the production and consumption of UPB could preserve environmental resources used in their production, while also delivering health benefits.

To the authors’ knowledge, no previous studies have investigated the environmental impacts of the broad category of UPB in Australia. Filling this knowledge gap is important as more countries consider environmental sustainability and include UPF terminology in their food policies^(20)^. Additionally, Australia lacks quantitative data on the environmental impacts of beverages, meaning limited information is available to inform reduction strategies. In this study, we aimed to quantify the environmental impacts of water-based UPB (UPB which contained water as an added ingredient) and bottled waters sold in Australia and assess the impacts of various scenarios which may reduce such impacts in the future.

## Methods

This study presents a quantitative environmental impact assessment based on data from existing peer-reviewed life-cycle assessment (LCA) datasets. We do not attempt to conduct an LCA.

### System boundaries and indicators

**Figure 1.**
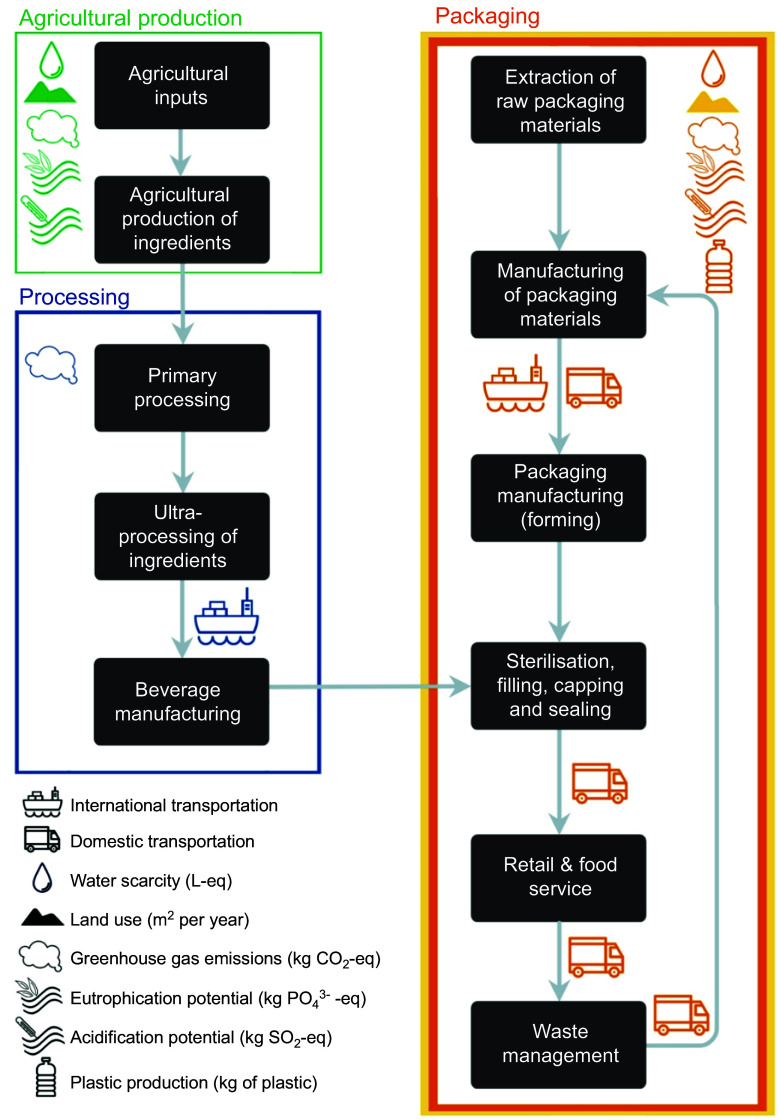
System boundaries for the analysis of the environmental impact of ultra-processed beverages and bottled water. Items in green indicate data from Poore & Nemecek (2018) database^(22)^, orange items indicate data from Warmerdam & Vickers^(23)^ and yellow items indicate data from PIQETv^(24)^. Blue items indicate data were sourced from the wider peer-reviewed literature and reports (see Supplementary Information for details). Note: ‘packaging’ includes primary, secondary and tertiary packaging. Secondary and tertiary packaging includes corrugated board, LDPE film and wooden pallets used to transport the bottles from the manufacturer to the retailer. Image created using Visme.

Figure 1 presents the UPB and bottled water system boundaries (i.e. lifecycle stages included in the analysis), adapted from the peer-reviewed literature^(7,18,21–23)^. The system is divided into four main stages:

1. Agricultural production of ingredients, including inputs (e.g. fertilisers and pesticides).
2. Primary, secondary and ultra-processing of ingredients and recombination of ingredients into the beverage.
3. International freight transportation of ingredients sourced from overseas based on the top-producing country (national transportation of ingredients was not calculated).
4. Extraction of raw packaging materials, manufacturing of packaging materials into preforms, bottle/ can manufacturing, sterilisation, filling, capping, sealing, transport to retail, transport of waste to landfill or recycling facility and end-of-life-related impacts.

Excluded items and the potential impacts of exclusions on study findings were investigated in detail and are outlined in the online supplementary material, Supplemental Information Section 1.

We chose common environmental impact indicators to represent the diversity of issues found in our previously developed model of the environmental impacts of UPF^(7)^ and for which data were available. Indicators used were greenhouse gas emissions (GHG, kg CO_2_-eq), land use (LU, km^2^ per year), eutrophication potential (EP, kg PO_4_^3–^eq), acidification potential (AP, kg SO_2_eq), water scarcity (WS, kL eq) and plastic use (kg) (Figure 1).

### Data

We used the Euromonitor Passport database to determine the current (2022) and projected (2027) sales of beverages in Australia and their accompanying ingredients^(25)^. Euromonitor data are sourced from trade associations, industry bodies, business press, company financial reports, company filings and official government statistics and has been widely used in food supply analyses^(26,27)^.

UPB (Asian specialty drinks, flavoured bottled water, carbonates, energy drinks, ready-to-drink tea, sports drinks and reconstituted juice) and non-UPB (carbonated bottled water, still bottled water), which included water as an added main ingredient were analysed. Euromonitor beverages were categorised as UPF or non-UPF based on the Euromonitor category names and definitions and cross-checked against a previous analysis of UPF which used the Euromonitor database^(28)^. We excluded alcoholic beverages, 100 % juices (non-reconstituted) and milks as water is not generally added during their supply chain. Additionally, milk and 100 % juices have nutritional contributions beyond hydration and thus comparing these products to tap water was not logical from a nutrition perspective. We also excluded non-dairy milk alternatives as the appropriate comparator from a dietary perspective (dairy milk) was excluded.

Drawing upon standard LCA practices, we applied a cut-off of 99 % of the total beverage by weight including packaging^(29)^. Items that were excluded by this rule tended to be ingredients for which there were no available environmental impact data, such as non-sugar sweeteners and other additives. Conversion factors for ingredients listed in the Euromonitor Database were applied to account for (1) the data being provided in weight instead of volume, (2) processing of the agricultural commodity or (3) the product requiring reconstitution prior to consumption (see online supplementary material, Supplemental Information File Section 3·2·2).

We sourced ingredient cradle-to-farmgate environmental impacts from a comprehensive meta-analysis of agricultural LCA containing standardised data from over 500 peer-reviewed publications^(22)^ (see Figure 1). Ingredient processing, ultra-processing, recombination in the beverage manufacturing facility and transportation data were sourced from peer-reviewed literature and reports. Where multiple data points were available, we inspected the original data and chose data points reflecting conventional production in the regions which produced the largest quantities of that food item. In instances where multiple data points were suitable, we chose an average impact (see online supplementary material, Supplemental Information Section 4·2·4).

We sourced packaging data from the Warmerdam and Vickers dataset, a recent industry commissioned and independently reviewed cradle-to-grave LCA on beverage packages available in Australian supermarkets in 2019/20^(23)^. This dataset contained GHG, EP, AP, WS, and plastic use data for cartons, polyethylene terephthalate (PET) bottles, recycled PET bottles, high-density polyethylene (HDPE) bottles, pouches, aluminium cans and glass bottles of varying sizes but did not contain any information about packaging-associated land use. Instead, we calculated land use by entering packaging sizes and types from the Warmerdam and Vickers dataset^(23)^ into LCA software, PIQET (Packaging Impact Quick Evaluation Tool^(24)^. PIQET was specifically designed to calculate the environmental impacts of packages in Australia^(24)^. Further details on the data are found in the online supplementary material, Supplemental Information, or original publications^(22–24)^.

Euromonitor Passport data and LCA data were matched manually in excel by the first author) and checked by a second author. We used consumer reports, FAOSTAT data and the scientific literature to determine the most likely match in instances where Euromonitor data were insufficiently disaggregated in comparison with the LCA data (see online supplementary material, Supplemental Information Section 4·2). For example, the Euromonitor ingredient ‘sugar’ was matched with sugar cane environmental data because sugarcane is responsible for the majority of sugar produced and utilised in Australia^(30)^. Where multiple ingredients were likely to be used, we took an average of the most common ingredients (see online supplementary material, Supplemental Information Section 3·2·1). Tea extract and plant extracts were excluded from the analysis due to limited environmental data.

Packaging types and sizes listed in the Euromonitor database were matched with the closest possible size and packaging type available in the Warmerdam & Vickers dataset, scaled to the actual product size. The weight of the PET bottles was not available in the Euromonitor database, but this information is important as weight of PET bottles substantially impact the related environmental impacts^(23)^. In order to address this, a supermarket audit was conducted to determine which products use lightweight *v*. regular strength PET plastic (see online supplementary material, Supplemental Information File Section 4·1).

Data were further extended by calculating GHG from international freight transportation based on the most common production centres for internationally produced ingredients (see online supplementary material, Supplemental Information File Section 5·2). We assumed that 100 % of each ingredient came from the country, which was the largest supplier of that ingredient to Australia.

### Data analysis

All analyses were based on the volume of beverages sold. The analysis was conducted in RStudio using cowplot, dplyr, ggplot2 and stringr.

#### Sales and packaging use

To retain the categorisations required to differentiate between UPB and non-UPB, we applied the proportions of packaging types to the sales categories and presented findings in bar charts. We combined the Euromonitor packaging data (2022 sales data) with an average packaging weight for each packaging type reported in Warmerdam and Vickers^(23)^. We further conducted a detailed market audit of the type of packaging used for non-carbonated beverages to differentiate products using lightweight *v*. regular weight PET packaging (carbonated beverages do not tend to be packaged in lightweight PET due to issues pertaining to carbonation retention). See online supplementary material, Supplemental Information Section 4·1 for further details.

#### Environmental impacts

Total environmental impacts were calculated, grouped by beverage type. For the intensities per litre calculations, we applied dilution factors, as recommended in LCA standards^(30)^.

#### Estimating the environmental impacts under different scenarios

We modelled four scenarios based on existing health and environmental targets (Table 1). The scenarios were based on real-world targets, and environmental savings were calculated for the year 2027 using sale projections from the Euromonitor Passport database relative to a 2022 baseline.

**Table 1.** Scenarios used to project the potential environmental impacts of Australian beverages in 2027 Scenarios were based on Euromonitor projections and modified according to existing global targets. Further descriptions are available in the Methods section

Scenario name	Description
2022 Baseline	Beverages sold in Australia in 2022, used as a baseline comparator for all other scenarios.
2027 Business as usual	2027 sales projections, assuming no changes in product formulation, consumption or recycling methods.
Reformulation only	Sugar and fruit juice concentrate levels reduced to achieve the WHO target of < 5 % total dietary energy from added sugars^(31)^.
20 % UPB reduction*	A 20 % reduction in the purchasing and consumption of UPB across all UPB categories, based on the French National target of 20 % UPF reduction^(32)^. Half of the avoided UPB were replaced with bottled water, the other half were replaced with tap water.
58 % recycled PET	58 % of PET bottles were replaced with recycled PET, based on the Australian Government’s national recycling target of 80 % recycled materials by 2030^(33)^.
Mixed approach	Combined approach modelling the collective impacts of reformulation, UPB reduction and improving recycling.

* Further reductions of 25 %, 50 %, 75 % and 100 % were also modelled to create online supplementary material, Supplemental Figure 4.

PET, UPB, ultra-processed beverages; UPF, ultra-processed beverages.

The first scenario was based on the WHO’s target for free sugar to contribute <5 % of total dietary energy^(31)^. We assumed this would be achieved through reformulation. Data on the total energy from added sugars from beverages were available from the most recent Australian Health Survey^(34)^, which enabled us to calculate the percent reductions in added sugars from products in our dataset to achieve the WHO target. We assumed that added sugars from food would also be reduced.

The second scenario was a 20 % reduction in the purchasing and consumption of UPB across all UPB categories, based on a French National target for reducing UPF consumption^(32)^. This target was announced in the 2019–2023 National Nutrition Target, by the French Ministry of Health. To our knowledge, this was the only quantifiable UPF target from any government. Because many dietary guidelines encourage avoidance of UPF^(20)^, we also modelled reductions in UPB consumption beyond the 20 % target.

We based the third scenario on the Australian Government’s 2030 ‘National Waste Policy Action Plan’, which resolved to achieve an 80 % recycling rate for all waste streams by 2030^(33)^. This was exclusively applied to the use of recycled PET bottles due to data limitations and justified because some of the other materials, such as aluminium, were already recycled at rates close to the target (aluminium recycling rates were 72 % in 2018)^(23)^. We applied linear interpolation to determine the proportional target for 2027, based on a baseline recycling rate of 12·6 % in the 2020–2021 financial year^(35)^. This resulted in the target of 58 % of PET bottles being made from recycled PET in 2027.

Finally, we created a ‘mixed approach’ that included all of the above targets to determine the impacts of a suite of policies. All scenarios were compared with the impacts of beverages in 2022, i.e. our baseline data.

## Results

### Sales and packaging use

**Figure 2.**
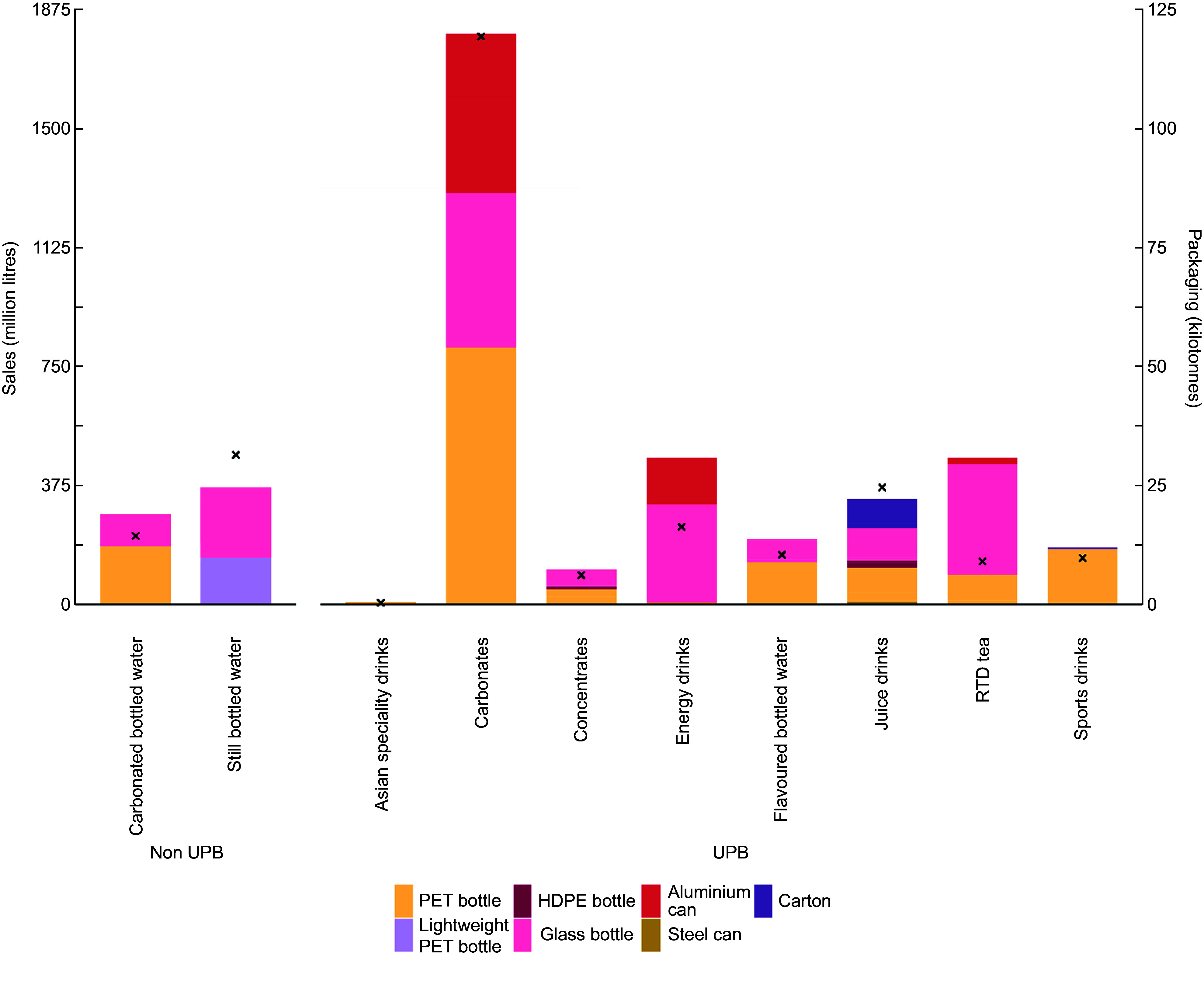
Total volume of beverages sold in Australia in 2022 and the associated quantity of consumer packaging. Results are displayed according to beverage category. ‘x’ indicate the sales (million litres sold in 2022), and bars refer to the packaging used (kilotonnes sold in 2022). RTD, ready-to-drink; UPB, ultra-processed beverage.

A total of 3623 million litres of beverages were sold across the Australian retail and food service sector in 2022 (Figure 2), with sales projected to rise by ∼180 million litres by 2027 (∼5 %) (see online supplementary material, Supplemental Figure S1). In 2022, UPB comprised 81 % of total sales by volume, with carbonates contributing the largest share of total sales (49 % of total sales by volume, Figure 2). The most common packaging type, in million litres sold, was PET bottles (50 %), followed by aluminium cans (28 %) and lightweight PET (28 %) (Figure 2).

### Environmental impacts of Australian water-based UPB compared with bottled waters in 2022

**Figure 3.**
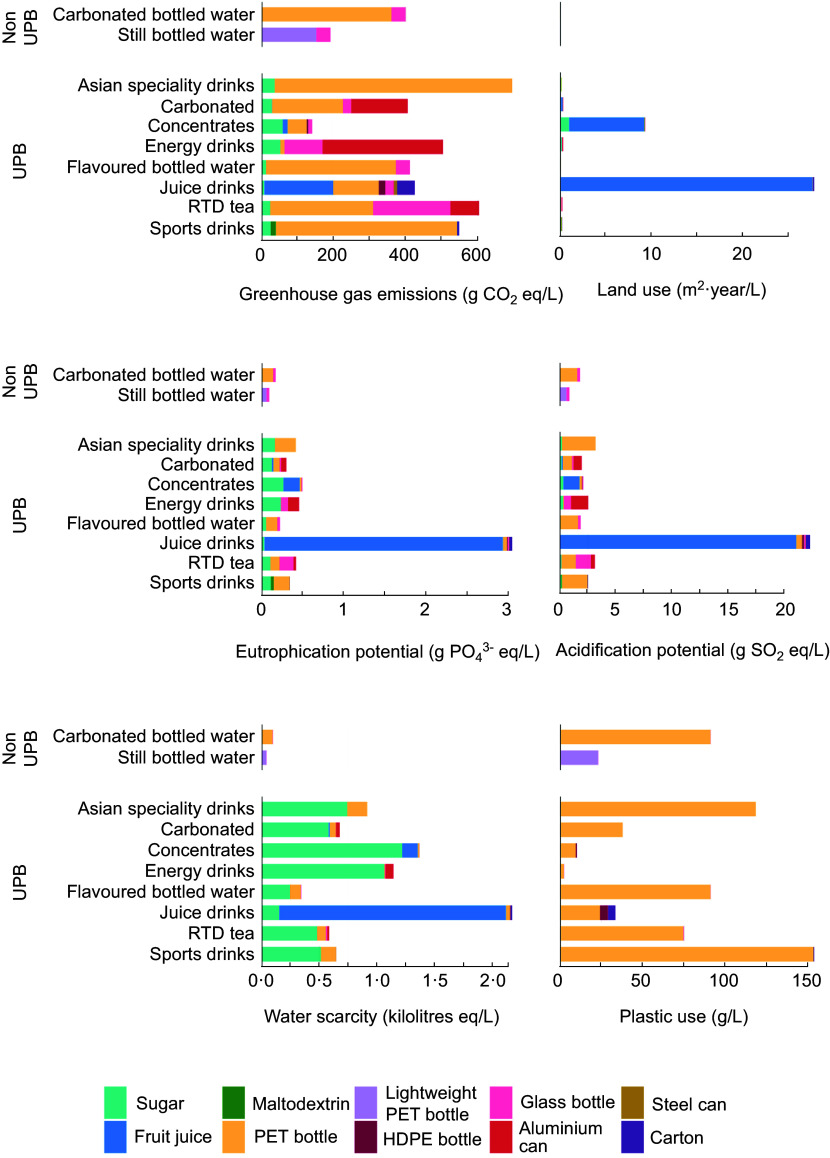
The environmental intensities of beverages sold in Australian in 2022 per litre of beverage content, grouped according to beverage categories, ingredients and packaging materials. Colours indicate the proportional impacts from the ingredients and packaging materials, both of which substantially contributed to environmental impacts. Greenhouse gas emissions are expressed in carbon dioxide equivalents per litre of beverage, land use is expressed in metres-squared per litre of beverage, eutrophication potential is expressed in phosphate equivalents per litre of beverage, acidification potential is expressed in sulphur dioxide equivalents per litre of beverage, water scarcity is expressed in kilolitres equivalent per litre of beverage and plastic use, which exclusively applies to the consumer packaging (i.e. it does not account for plastic used throughout the packaging or ingredient supply chains), is measured in grams per litre of beverage. RTD, ready-to-drink; UPB, ultra-processed beverage.

Our analysis revealed that on a per litre basis, non-UPB generally had lower impacts compared with UPB. The exception to this was plastic use where carbonated bottled water ranked third highest in overall contribution to plastic (Figure 3; see online supplementary material, Supplemental Figure S2). Impacts from still bottled water remained low due to the use of lightweight PET.

**Figure 4.**
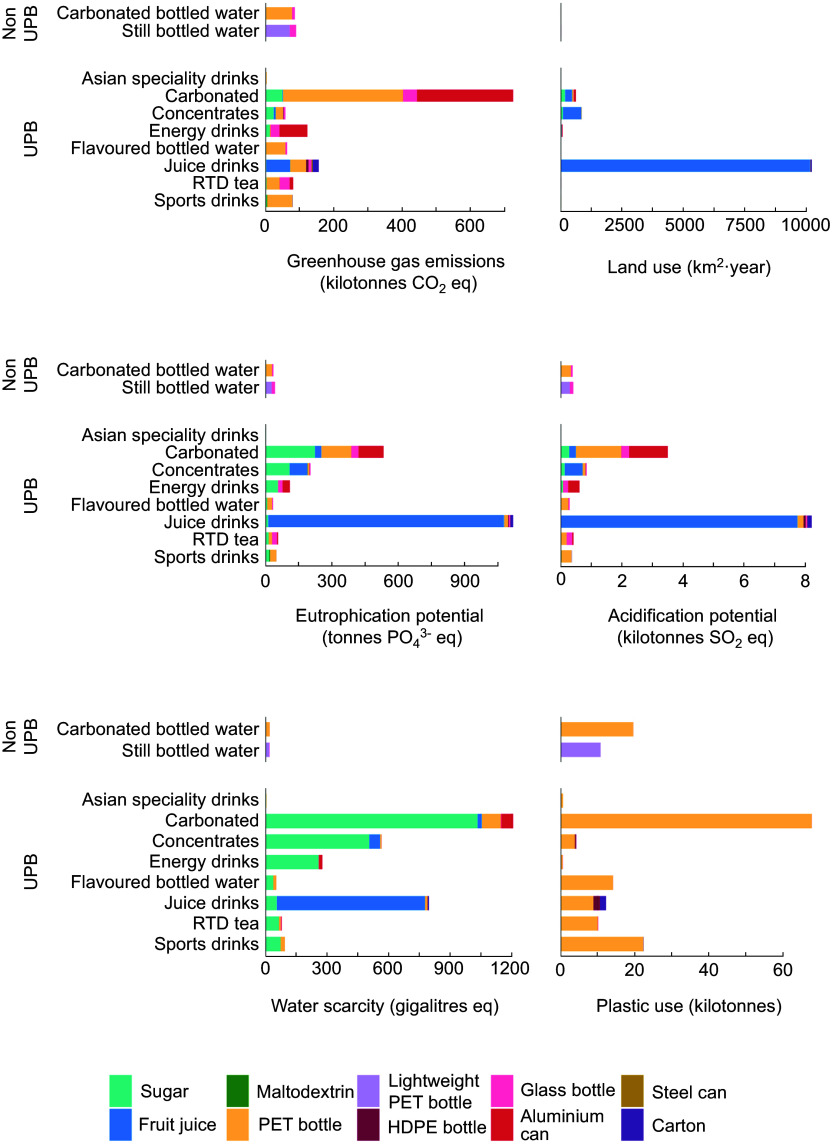
The environmental impacts associated with the total volume of beverages sold in Australia in 2022, grouped according to beverage categories, ingredients and packaging materials. Colours indicate the proportional impacts from the ingredients and packaging materials, both of which substantially contributed to environmental impacts. Greenhouse gas emissions are expressed in carbon dioxide equivalents, land use is expressed in metres-squared, eutrophication potential is expressed in phosphate equivalents, acidification potential is expressed in sulphur dioxide equivalents, water scarcity is expressed in kilolitres equivalent and plastic use, which exclusively applies to the consumer packaging (i.e. it does not account for plastic used throughout the packaging or ingredient supply chains), is measured in grams. RTD, ready-to-drink; UPB, ultra-processed beverage.

When impacts were estimated on the basis of 2022 sales, it was found that UPB accounted for the majority of aggregate impacts from beverages in 2022 (88 % of GHG, 95 % of AP, 96 % of EP, 98 % of WS, 99·6 % of LU and 81 % of plastic use; see online supplementary material, Supplemental Table S6). Carbonates contributed the most to GHG, WS and plastic, whereas juice drinks accounted for the largest proportion of LU, AP and EP (Figure 4).

GHG from beverages were primarily driven by packaging (53–100 % of impacts per total sales in 2022, depending on the beverage) (see online supplementary material, Supplemental Figure S2 & S3), with plastic packaging (PET, lightweight PET and HDPE) contributing the most to GHG (see online supplementary material, Supplemental Figure S2). Conversely, LU, AP, EP and WS were driven by ingredients, with packaging playing a smaller role (see online supplementary material, Supplemental Figure S2 & S3). Plastic use impacts are exclusively derived from the packaging stage.

Online supplementary material, Supplemental Figure S3 shows impacts according to the supply chain stage and highlights the dominant role of the agricultural production stage, particularly in LU and WS (see online supplementary material, Supplemental Table S6). These impacts were driven by the juice drinks category due to the reliance of these products on fruit juice concentrate (Figure 4), which had a higher environmental intensity relative to other ingredients. Sugar also made a substantial contribution to WS footprints among UPB.

### Environmental mitigation potential under different scenarios

**Figure 5.**
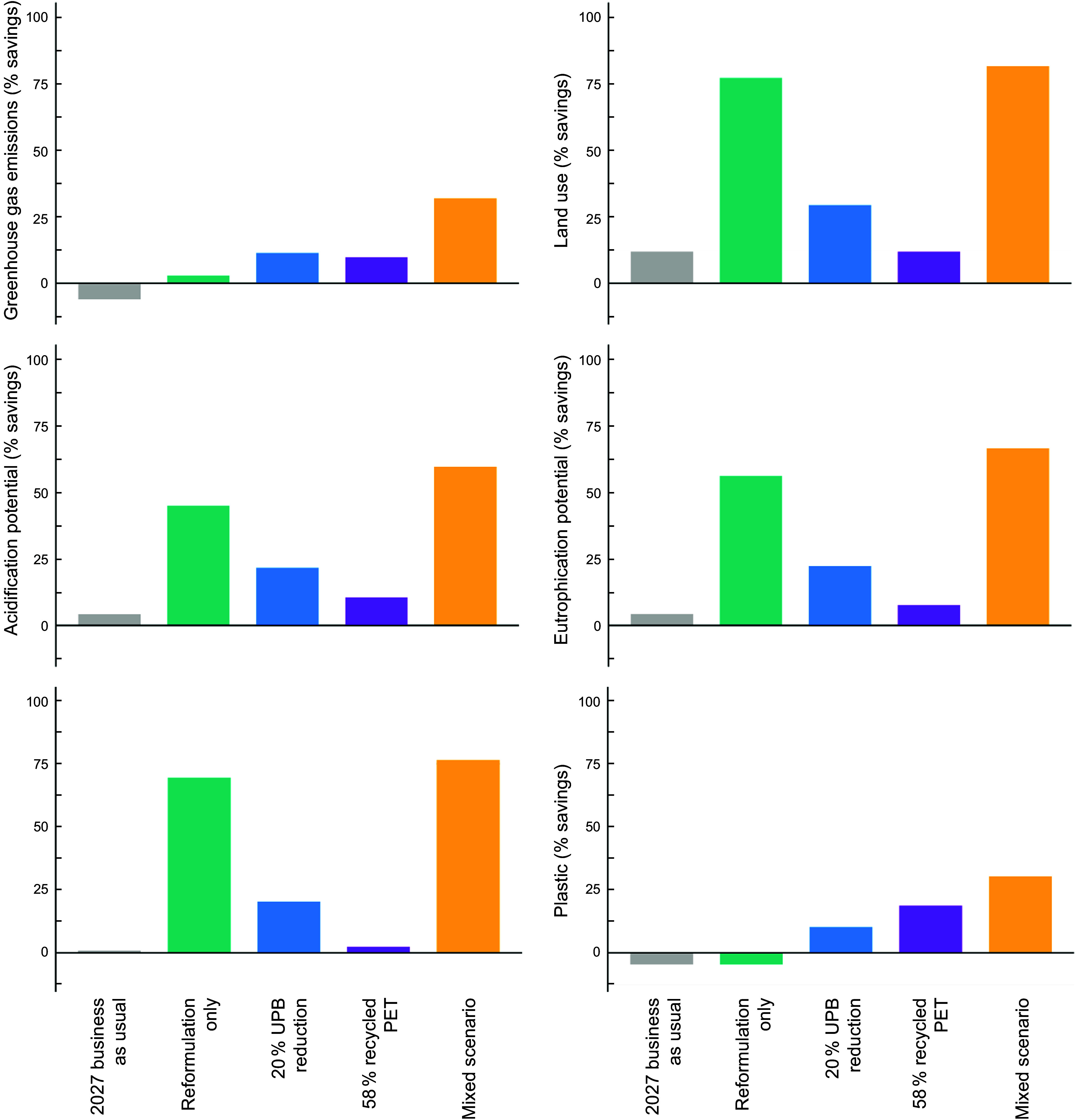
Modelled environmental impacts of water-based beverages sold in Australia under different policy-based scenarios based on sales projections to 2027. All results are presented as percentage environmental savings compared with a 2022 baseline. Scenario descriptions are found in Table 1. PET, polyethylene terephthalate.

Our modelling suggests that inaction between 2022 and 2027 would result in a 6 % increase in GHG and 5 % increase in plastic use associated with beverages (Figure 5). However, based on market predictions, AP, EP, LU and WS show a slight reduction (1–12 %) decrease by 2027 owing to a projected 13 % decrease in juice drink sales, which makes a significant aggregate contribution across these indicators (see online supplementary material, Supplemental Table S7).

The reformulation strategy resulted in limited GHG savings (3 %) and increased plastic use (by 5 %). However, reformulation resulted in the largest improvements for LU, AP, EP and WS from any single strategy (45–77 % reductions in impacts, see online supplementary material, Supplemental Table S7). This was driven by reductions in agricultural production of sugar and fruit juice concentrate. Reducing UPB by 20 % was moderately effective for all indicators (10–29 % reductions in impacts). Online supplementary material, Supplemental Figure S4 & Table S7 indicate that more ambitious reductions in UPB led to corresponding reductions in environmental impacts, with the most substantial savings associated with avoiding UPB altogether. Increasing the use of recycled PET plastics was also moderately effective across all indicators (2–19 % reductions in impacts).

The ‘mixed approach’ resulted in the greatest environmental savings (30–82 % reductions in impacts), compared with any other individual scenario. The ‘mixed approach’ scenario resulted in a 32 % reduction in GHG, 60 % reduction in AP, 67 % reduction in EP, 77 % reduction in WS, 82 % reduction in LU and 30 % reduction in plastic use (see online supplementary material, Supplemental Table S7).

## Discussion

This study analysed the environmental impacts of water-based UPB and bottled waters in Australia using six key environmental indicators. Our results indicate that UPB are associated with higher environmental impacts than bottled waters. Specifically, carbonates (i.e. soft drinks) were a key driver of greenhouse gas emissions, water scarcity and plastic use. Carbonates also made the largest contribution to many of the environmental impacts according to total sales in 2022, and projected 2027 sales, even though they did not have the highest intensities per litre. This highlights the importance of considering impacts according to sales or consumption patterns, as ultimately the products with the highest impacts are likely to be those that are frequently purchased and consumed, as found previously^(36)^. In contrast, juice drinks had the highest environmental intensities per litre and also by sales when considering land use, acidification and eutrophication potential, which can be attributed to the agricultural production of fruit (see online supplementary material, Supplemental Table S7).

All scenarios modelled in this study resulted in meaningful reductions in environmental impacts. The reformulation strategy we modelled suggested a successful reduction in impacts for most indicators, driven by the removal of substantial quantities of added sugar and fruit juice concentrate. However, it is critical to note that we did not model any replacements for these ingredients as they comprised < 1 % of the total beverage and had no available LCA data. In reality, reformulation strategies tend to result in increased use of non-sugar sweeteners^(27)^, which are also associated with negative health impacts^(12)^, and are likely to also have some environmental impacts^(37)^. Indeed, addressing food system issues using reformulation alone has been criticised for being reductionist^(38)^ as this strategy may fail to address the broader issues driving the sales and consumption of superfluous foods and beverages. Further, reformulation is unlikely to promote dietary shifts and thus may not assist in achieving the recommendations set out by food-based dietary guidelines, which in this case is to choose tap water^(39)^.

Reductions in UPB consumption to meet the target of a 20 % reduction in UPF purchasing and consumption resulted in modest environmental benefits across all indicators. We also modelled reductions in UPB beyond the 20 % target, with environmental benefits more or less proportionate to UPB reductions. Indeed, a hypothetical 75 % reduction of UPB outperformed the ambitious reformulation target modelled in this study for all indicators except for land use where an equal benefit was found.

In the scenarios where UPB reductions were modelled, we assumed that half of the UPB avoided would be replaced by bottled water and the other half replaced by tap water, given tap water is generally regarded as safe to drink in Australia^(40)^. Evidence suggests the environmental impacts of tap water are substantially lower than bottled water^(17–19)^, even when it relies on high energy-consuming filtration technologies such as desalination^(18)^. Switching to tap water could help meet international plastic reduction agreements^(41)^, as well as deliver health^(42)^ and economic benefits^(19)^. However, consumers who prefer to drink bottled water for taste, convenience or due to safety concerns^(40)^ may need to be convinced of this change. This may be possible given approximately 80 % of Australian consumers would consider reducing their purchases of bottled water for sustainability reasons^(43)^.

The packaging scenario we modelled in this study focused exclusively on improving the use of recycled PET, one of Australia’s most common beverage packaging materials (Figure 1). Existing rates of recycling PET bottles in Australia are low (12·6 % of Australian plastics recycled in 2020–2021)^(35)^, and falling^(44)^, decreasing the chance of meeting the ambitious target of 80 % recycling by 2030^(33)^. However, a potential upturn in Australian PET bottle recycling rates is likely over the next few years due to the construction of new PET recycling facilities, which are proposed to recycle the equivalent of 1 billion 600 ml PET bottles annually^(45)^.

The final ‘mixed’ scenario demonstrated the highest potential savings due to cumulative reductions in impacts from each scenario. This finding aligns with previous studies suggesting that meaningful reductions in food system environmental impacts are likely to require concurrent shifts in consumption, production methods and waste management^(46)^, all of which were modified in the ‘mixed’ scenario. Ultimately, combining impact reduction methods and thus making changes across the system resulted in the most benefit.

Strengths of our study included the use of peer-reviewed data sources to compile a comprehensive database of country-specific environmental intensities for each relevant unit process (see online supplementary material, Supplemental Information for full details), alignment with core lifecycle assessment principles and the focus on overall sales, rather than comparisons based solely on a per litre basis. The latter factor was important to measure the real-world impacts of Australian beverages, rather than a hypothetical comparison between products. As the Euromonitor dataset is global, this study provides an approach that could be replicated to estimate the impacts of UPB in other geographies.

Another key strength was the use of multiple indicators in the analysis which were purposely chosen to represent a wide variety of environmental impacts. Previous research demonstrates that the metrics used to quantify environmental impacts in this study provide a comprehensive view of broader environmental issues and are likely to be associated with flow-on impacts for other environmental issues, such as biodiversity loss and soil degradation^(7)^. As a result, this study enables a relatively holistic view of environmental sustainability issues.

Despite these strengths, some limitations exist. Methodological assumptions and heterogeneous data sources potentially create uncertainty around our estimates. Key sources of uncertainty include transportation impacts (only international transportation was measured, and all commodities were assumed to originate from the top producing country), included ingredients (e.g. juice values were based on the juice flavours with highest sales; orange and apple). Uncertainties likely also arose from dataset matching. For example, packaging impacts differed based on the size of the package, although we addressed this by scaling packaging impacts according to package size. Further, we did not measure all supply chain stages, such as refrigeration, due to limited data availability (see online supplementary material, Supplemental Information Section 1).

Our study included beverages that had water added during their production lifecycles and thus excluded 100 % fruit juices, milk-based beverages and alcoholic beverages. The impacts of tap water were not included in the analysis due a lack of comparable data. This is justified because water was found in all the included beverages, the environmental impacts of tap water were assumed to be nullified when implementing a comparative assessment. If other beverages were included environmental impacts from both UPB and non-UPB would likely have been higher, and savings resulting from switching from UPB to non-UPB may not have been as apparent. For example, plant-based milks, which are often UPB, tend to have lower environmental impacts than dairy milks^(47)^.

Despite these limitations, our findings are aligned with previous studies. Specifically, the greenhouse gas emissions and water scarcity values estimated here align with studies from the UK and Europe^(17,19,21,48,49)^. At the time of writing, only one other study had been published on acidification and eutrophication impacts of beverages, and estimates differed significantly from those presented here^(21)^, most likely because acidification and eutrophication are highly region-dependent. No comparable published estimates of land or plastic use were identified.

### Conclusion

This study found that the environmental impacts of water-based UPB are significantly larger than bottled waters across all key environmental indicators in Australia. Scenarios to reduce environmental impacts based on relevant health and recycling targets were modelled. The largest potential environmental savings were found when a combination of strategies from diverse policy portfolios were used to reduce overall consumption of UPB, reduce the use of UPB ingredients (i.e. reformulation) and utilise recycled PET bottles. Findings demonstrate that policies may be more effective when they are implemented in a suite of complementary strategies.

## Supporting information

Anastasiou et al. supplementary material 1 Anastasiou et al. supplementary material

Anastasiou et al. supplementary material 2 Anastasiou et al. supplementary material

## Supplementary material

For supplementary material accompanying this paper visit https://doi.org/10.1017/S1368980025000187

## References

[1] Chen C , Chaudhary A & Mathys A (2022) Dietary change and global sustainable development goals. Front Sustain Food Syst 6, 771041.

[2] Rockström J , Edenhofer O , Gaertner J et al. (2020) Planet-proofing the global food system. Nat Food 1, 3–5.

[3] Clark MA , Domingo NGG , Colgan K et al. (2020) Global food system emissions could preclude achieving the 1·5° and 2°C climate change targets. Science 370, 705–708. 33154139 10.1126/science.aba7357

[4] Goessler CLJ , Liu M , McClure E et al. (2023) Reshaping Australian Food Systems: A Roadmap Towards a More Sustainable, Productive and Resilient Future for Australia’s Food, its Environment and People. Canberra: CSIRO.

[5] Australian Institute of Health and Welfare (2021) Australian Burden of Disease Study 2018: Key Findings. Canberra: AIHW, Australian Government.

[6] Anastasiou K , Baker P , Hadjikakou M et al. (2022) A conceptual framework for understanding the environmental impacts of ultra-processed foods and implications for sustainable food systems. J Cleaner Prod 368, 133155.

[7] Anastasiou K , Baker P , Hendrie GA et al. (2023) Conceptualising the drivers of ultra-processed food production and consumption and their environmental impacts: a group model-building exercise. Global Food Secur 37, 100688.

[8] Fardet A & Rock E (2020) Ultra-processed foods and food system sustainability: what are the links? Sustainability 12, 6280.

[9] Monteiro C , Cannon G , Levy R et al. (2019) Ultra-processed foods: what they are and how to identify them. Public Health Nutr 22, 936–941. 30744710 10.1017/S1368980018003762 PMC10260459

[10] Lane MM , Gamage E , Du S et al. (2024) Ultra-processed food exposure and adverse health outcomes: umbrella review of epidemiological meta-analyses. BMJ 384, e077310. 38418082 10.1136/bmj-2023-077310 PMC10899807

[11] Malik VS & Hu FB (2022) The role of sugar-sweetened beverages in the global epidemics of obesity and chronic diseases. Nat Rev Endocrinol 18, 205–218. 35064240 10.1038/s41574-021-00627-6 PMC8778490

[12] World Health Organization (2023) Use of Non-Sugar Sweeteners: WHO Guideline. Geneva, Switzerland: World Health Organization.

[13] Machado PP , Steele EM , Louzada ML et al. (2020) Ultra-processed food consumption drives excessive free sugar intake among all age groups in Australia. Eur J Nutr 59, 2783–2792. 31676952 10.1007/s00394-019-02125-y

[14] DellaValle DM , Roe LS & Rolls BJ (2005) Does the consumption of caloric and non-caloric beverages with a meal affect energy intake? Appetite 44, 187–193. 15808893 10.1016/j.appet.2004.11.003

[15] Cámara M , Giner RM , González-Fandos E et al. (2021) Food-Based dietary guidelines around the world: a comparative analysis to update AESAN Scientific Committee Dietary Recommendations. Nutrients 13, 3131. doi: 10.3390/nu13093131. 34579007 PMC8471688

[16] Australian Bureau of Statistics 2020–21 Apparent Consumption of Selected Foodstuffs, Australia. (2022) https://www.abs.gov.au/statistics/health/health-conditions-and-risks/apparent-consumption-selected-foodstuffs-australia/latest-release (accessed 08 June 2023).

[17] Hanssen OJ , Rukke E-O , Saugen B et al. (2007) The environmental effectiveness of the beverage sector in Norway in a Factor 10 perspective. Int J Life Cycle Assess 12, 257.

[18] Fantin V , Scalbi S , Ottaviano G et al. (2014) A method for improving reliability and relevance of LCA reviews: the case of life-cycle greenhouse gas emissions of tap and bottled water. Sci Total Environ 476–477, 228–241. 24463258 10.1016/j.scitotenv.2013.12.115

[19] Botto S , Niccolucci V , Rugani B et al. (2011) Towards lower carbon footprint patterns of consumption: the case of drinking water in Italy. Environ Sci Policy 14, 388–395.

[20] Anastasiou K , Ribeiro De Melo P , Slater S et al. (2022) From harmful nutrients to ultra-processed foods: exploring shifts in ‘foods to limit’ terminology used in national food-based dietary guidelines. Public Health Nutr 26(11), 1–12. 36458692 10.1017/S1368980022002580 PMC10641640

[21] Amienyo D , Gujba H , Stichnothe H et al. (2013) Life cycle environmental impacts of carbonated soft drinks. Int J Life Cycle Assess 18, 77–92.

[22] Poore J & Nemecek T (2018) Reducing food’s environmental impacts through producers and consumers. Science 360, 987. 29853680 10.1126/science.aaq0216

[23] Warmerdam S & Vickers J (2021) LCA of Beverage and Food Packaging in Australia and New Zealand. Wellington, New Zealand: thinkstep Ltd, prepared for Tetra Pak Oceania.

[24] Verghese K , Horne R & Carre A (2010) PIQET: the design and development of an online streamlined LCA tool for sustainable packaging design decision support. Int J Life Cycle Assess 15, 608–620.

[25] Euromonitor (2023) Market Analysis: Our Methodology. https://www.euromonitor.com/our-methodologies/market-analysis (accessed 28 April 2023).

[26] Popkin BM & Reardon T (2018) Obesity and the food system transformation in Latin America. Obes Rev 19, 1028–1064. 29691969 10.1111/obr.12694 PMC6103889

[27] Russell C , Baker P , Grimes C et al. (2022) Global trends in added sugars and non-nutritive sweetener use in the packaged food supply: drivers and implications for public health. Public Health Nutr 26(5), 1–13. 35899782 10.1017/S1368980022001598 PMC10346066

[28] Baker P , Machado P , Santos T et al. (2020) Ultra-processed foods and the nutrition transition: global, regional and national trends, food systems transformations and political economy drivers. *Obes Rev 21*, e13126. 10.1111/obr.13126 32761763

[29] Environmental Product Declarations (2022) Product Category Rules: Soft Drinks, vol. PRODUCT CATEGORY CLASSIFICATION: UN CPC 24490, pp. 9–10. https://www.environdec.com/pcr-library (accessed 20 April 2023).

[30] FSANZ (2019) Sugar. https://www.foodstandards.gov.au/consumer/nutrition/Pages/Sugar.aspx (accessed 26 April 2023).

[31] World Health Organization (2015) Guideline: Sugars Intake for Adults and Children. Geneva: World Health Organization. 25905159

[32] Ministere de la Santa et de la Prevention (2018) *Programme National Nutrition Santé 2019–2023 [Translated to English]*. Ministere de la Santa et de la Prevention. https://www.environdec.com/pcr-library (accessed 21 August 2023).

[33] Australian Government (2019) National Waste Policy Action Plan 2019. Canberra, Australia: Australian Government.

[34] Australian Bureau of Statistics (2016) 4364.0.55.011 - Australian Health Survey: Consumption of Added Sugars, 2011–2012. https://www.abs.gov.au/ausstats/abs@.nsf/lookup/4364.0.55.011main+features12011-12 (accessed 16 November 2023).

[35] O’Farrell K , Harney F & Stovell L (2022) Australian Plastics Flows and Fates Study 2020–21 – National Report. no. P1348. Canberra, Australia: Department of Climate Change, Energy, the Environment and Water.

[36] Ridoutt BG , Anastasiou K , Baird D et al. (2020) Cropland footprints of Australian dietary choices. Nutrients 12, 1212. 32344857 10.3390/nu12051212 PMC7282022

[37] Praveena SM , Cheema MS & Guo H-R (2019) Non-nutritive artificial sweeteners as an emerging contaminant in environment: a global review and risks perspectives. Ecotoxicol Environ Saf 170, 699–707. 30580164 10.1016/j.ecoenv.2018.12.048

[38] Scrinis G & Monteiro CA (2018) Ultra-processed foods and the limits of product reformulation. Public Health Nutr 21, 247–252. 28703086 10.1017/S1368980017001392 PMC10261094

[39] NHMRC (2013) Eat For Health Educator Guide. Canberra, Australia: Commonwealth of Australia.

[40] Ragusa AT & Crampton A (2016) To buy or not to buy? Perceptions of bottled drinking water in Australia and New Zealand. Hum Ecol 44, 565–576.

[41] United Nations Environment Programme (2023) Turning off the Tap: How the World Can End Plastic Pollution and Create a Circular Economy. Nairobi: United Nations Environment Programme. ISBN: 978–92–807–4024–0.

[42] World Health Organization (2022) WHO Manual on Sugar-Sweetened Beverage Taxation Policies to Promote Healthy Diets. Geneva: World Health Organization.

[43] Pearson D , Friel S & Lawrence M (2014) Building environmentally sustainable food systems on informed citizen choices: evidence from Australia. Biol Agric Hortic 30, 183–197.

[44] Australian Packaging Covenant (2021) APCO Collective Impact Report. Sydney, Australia: Australian Packaging Covenant Organisation.

[45] Circulate Plastics Australia (2023) Australasia’s Largest End-to-End PET Recycling Facilities. https://circularplasticsaustralia.com/our-projects/pet/ (accessed 09 November 2023).

[46] Springmann M , Clark M , Mason-D’Croz D et al. (2018) Options for keeping the food system within environmental limits. Nature 562, 519–525. 30305731 10.1038/s41586-018-0594-0

[47] Carlsson Kanyama A , Hedin B & Katzeff C (2021) Differences in environmental impact between plant-based alternatives to dairy and dairy products: a systematic literature review. Sustainability 13, 12599.

[48] Boesen S , Bey N & Niero M (2019) Environmental sustainability of liquid food packaging: is there a gap between Danish consumers’ perception and learnings from life cycle assessment? J Cleaner Prod 210, 1193–1206.

[49] Niccolucci V , Rugani B , Botto S et al. (2010) An integrated footprint based approach for environmental labelling of products: the case of drinking bottled water. Int J Des Nat Ecodynamics 5, 68–75.

