# A quantitative environmental impact assessment of Australian ultra-processed beverages and impact reduction scenarios – ERRATUM

**DOI:** 10.1017/S1368980025000497

**Published:** 2025-05-02

**Authors:** Kim Anastasiou, Michalis Hadjikakou, Ozge Geyik, Gilly A Hendrie, Phillip Baker, Richard Pinter, Mark Lawrence

Cambridge University Press apologise for an error in the labelling of the y-axis of Figure 5 in the above article. The correct version of the figure is below.

**Published Figure**:

**Figure 5.**
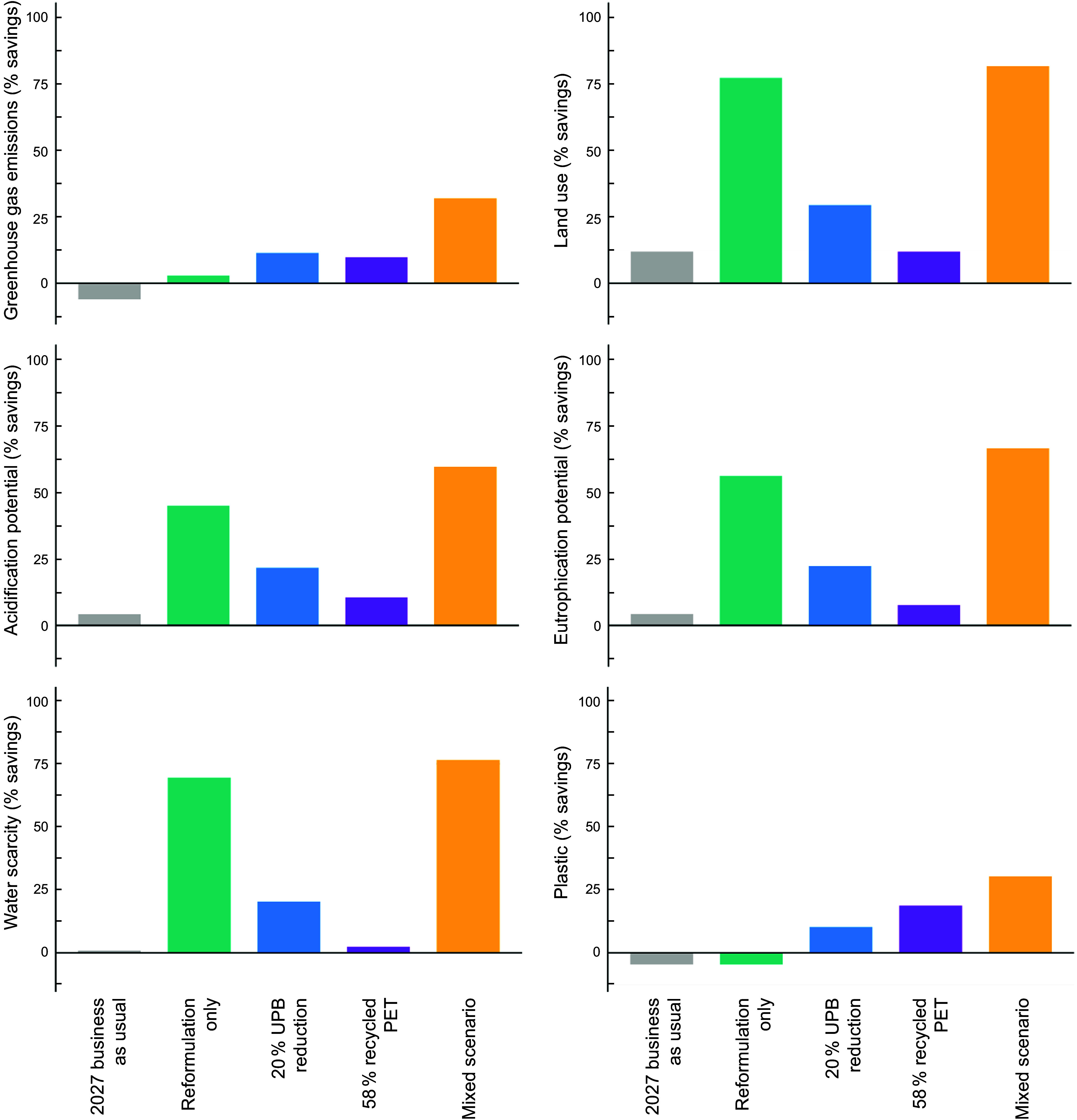
Modelled environmental impacts of water-based beverages sold in Australia under different policy-based scenarios based on sales projections to 2027. All results are presented as percentage environmental savings compared with a 2022 baseline. Scenario descriptions are found in Table 1. PET , polyethylene terephthalate.

**Correct Figure**:

**Figure 5.**
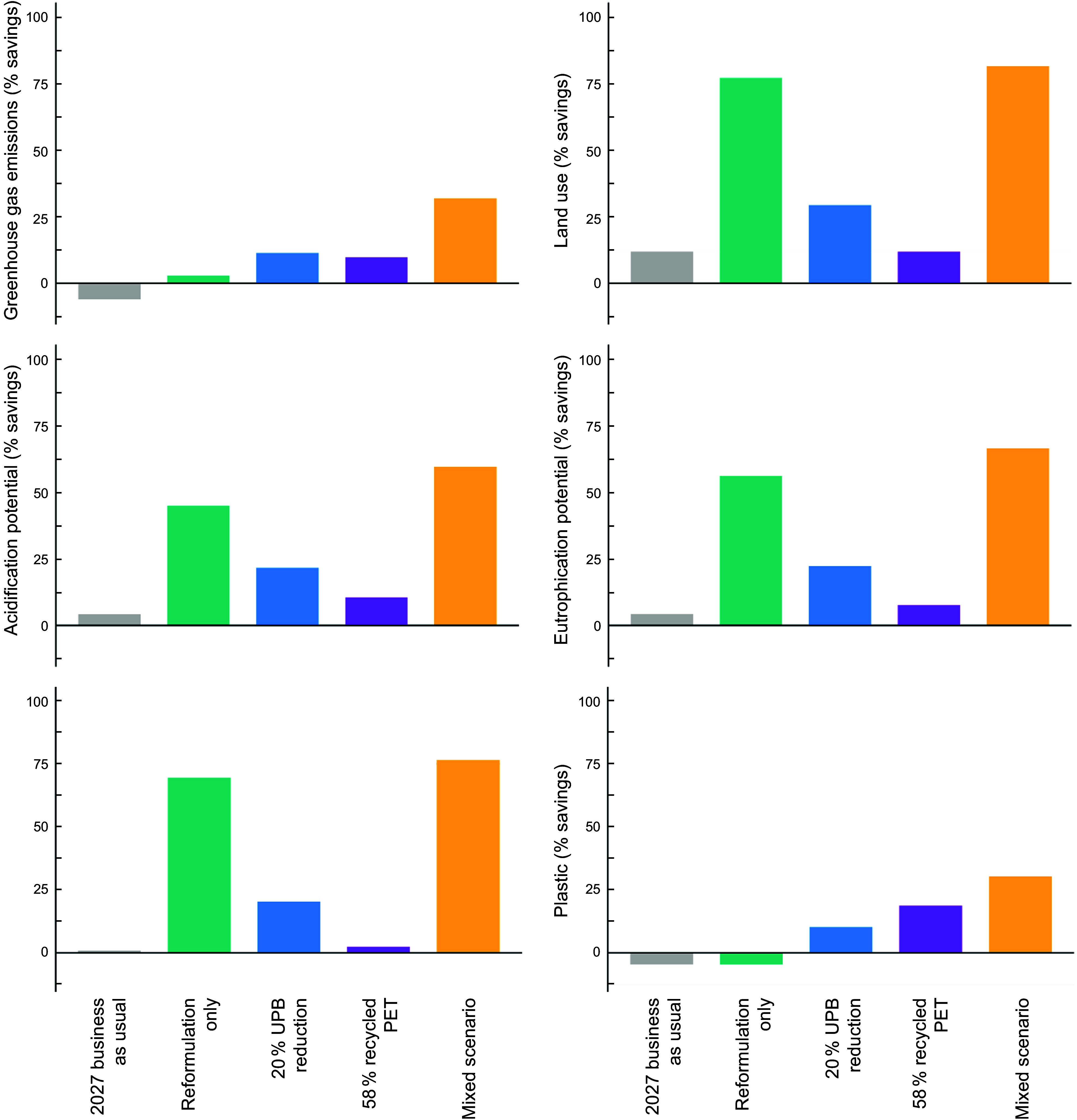
Modelled environmental impacts of water-based beverages sold in Australia under different policy-based scenarios based on sales projections to 2027. All results are presented as percentage environmental savings compared with a 2022 baseline. Scenario descriptions are found in Table 1. PET , polyethylene terephthalate.
