# Supplementary material for: A quantitative environmental impact assessment of Australian ultra-processed beverages and impact reduction scenarios
Source: Public Health Nutr. 2025 Feb 4;28(1):e51. doi: 10.1017/S1368980025000187 (PMC11984005; doi:10.1017/S1368980025000187)
Supplement: Anastasiou et al. supplementary material 2 — Anastasiou et al. supplementary material [file S1368980025000187sup002.pdf]

Supplementary Information File

The following file contains detailed information regarding the methods used in the study Anastasiou K, Hadjikakou M, Geyik O, Hendrie G, Baker P, Pinter R & Lawrence M (2024) *"A quantitative environmental impact assessment of Australian ultra-processed beverages and impact reduction scenarios"*

Contents

1 Determining system boundaries 2

2 Environmental indicators 5

3 Preparing data from Euromonitor 6

3.1 Packaging dataset 6

3.2 Ingredient and sales data 7

3.2.1 Determining inclusion of ingredients 7

3.2.2 Ingredient conversion factors 7

4 Matching datasets 9

4.1 Packaging data 9

4.2 Matching ingredient data 13

4.2.1 Main assumptions 13

4.2.2 Assumptions regarding the commodity chosen for inclusion 13

4.2.3 Assumptions regarding the country of origin of ingredients 14

4.2.4 Final decisions regarding the specific data point included in the analysis 14

5 Sourcing additional data 15

5.1 Ingredient and beverage manufacturing 15

5.2 Transportation of ingredients 16

6 Supplementary Tables and Figures 17

6.1 Supplementary Sales Figures 17

6.2 Supplementary Environmental Impact Figures and Tables 18

7 References 25

1 Determining system boundaries

System boundaries were adapted from our previously published model which displays the environmental impacts of ultra-processed foods [1], with product-specific details added based on system boundary diagrams published in the peer-reviewed literature [2-4].

We subsequently mapped the information available from the two main datasets used in the analysis [5, 6] to the system boundaries diagram. We considered and subsequently excluded items described in Table S1, predominantly due to a lack of rigorous data and thus the need to rely on potentially erroneous assumptions to calculate the values. The final system boundaries are displayed in Figure 1 in the main article and described below.

The system is divided into four main stages, as follows:

1. Agricultural production: agricultural production of beverage ingredients (other than water), including inputs such as fertilisers and pesticides.
2. Processing: primary, secondary and ultra-processing of ingredients, recombination of ingredients at the beverage manufacturing facility into the final beverage.
3. Transport: international freight transport of ingredients sourced from overseas.
4. Packaging production: extraction of raw materials used for the production of packaging, manufacturing of packaging materials into glass pellets, PET preforms, aluminium sheets etc, transport to the bottle/ can forming facility, manufacture into the final packaging product, sterilisation of bottles, filling, capping and sealing, transport to retail, transport of waste to landfill or recycling facility, end-of-life-related impacts.

Table S1: Supply chain stages, packaging types and ingredients excluded from analysis, including the potential impact on findings.

Supply chain stage or item excluded	Potential impact on findings	
	**Switch from UPFs to bottled water**	**Switch from UPFs & bottled water to tap water**
Refrigeration	Both beverages classified as UPFs and bottled water are likely to be refrigerated and therefore similar factors are likely to be applied, effectively cancelling each other out.	Previous studies have reported that refrigeration has limited impact on final results [5, 7], although one study did find that refrigeration had an impact on the study outcomes [2]
Food loss and waste	May underestimate impacts of ingredients included in UPFs.	
Domestic transport of ingredients prior to beverage manufacturing	Unlikely to have substantial impact because both spring water and ingredients in UPFs are transported domestically.	May underestimate the environmental gains from switching to tap water.
Wastewater treatment processes	May underestimate the environmental gains from switching to bottled water as sugar-sweetened beverage processing plants have been reported to have wastewater issues [8].	Wastewater treatment from water filtration practices would not have substantial impacts as all products were assumed to contain filtered water.
Off-shore recycling of plastic, paper or card-based packaging	May underestimate the environmental gains from switching to bottled water.	May underestimate the environmental gains from switching to tap water.
Processes associated with water production	We assumed that tap water is used in the production of most of the beverages included in our analysis and therefore this would not impact the differences in environmental impacts between the beverage categories.	
Recycled PET bottles	100% recycled PET bottles were excluded from the main analysis as we had no way of determining the proportion of products using recycled material, and because plastic recycling rates in Australia are low (13% in 2021) [9]. Impacts on the outcomes of the comparison between bottled water and UPFs are likely limited because both categories use PET bottles.	Potentially substantial impacts, hence our modelling of this scenario in the final section of the analysis.

Supply chain stage or item excluded	Potential impact on findings	
	Switch from UPFs to bottled water	Switch from UPFs & bottled water to tap water
Ingredients which comprised <1% of total product by volume	Excluded ingredients were exclusive to the UPF category and thus the scenarios modelling switches to bottled or tap water may underestimate environmental benefits.	
Supply chain stages included in analyses for acidification potential, eutrophication potential, land use and water scarcity were limited to ingredient production and packaging production only	Previous research on the environmental impacts of beverages have indicated that the majority of impacts for acidification potential, eutrophication potential, and water-related impacts are limited to the production of the ingredients and the packaging [2, 10]. It was expected that diet-related land use stems predominantly from agricultural production [11]. It was expected that these exclusions would not substantially impact the results.	
Pouches, bulk bottled waters or 4L or more.	These data were excluded due to a lack of packaging data. We were unable to source land use data for pouches, and bulk bottled waters of 4L or more, which were commonly packaged in 'bag in boxes'. These data accounted for 3% of the total sales in 2022 and 3% of the total projected sales in 2027. Breakdowns of the percent of total products excluded per category, per year follow. Still bottled water: 15% (2022), 16% (2027); Juice drinks 3% (2022), 4% (2027); Sports drinks 2% (2022), 3% (2027).	

2 Environmental indicators

Environmental indicators included in our analysis are outlined below.

Table S2: Indicators used in the analysis.

Indicator	Description	Units (may differ in figures)
Land use	Land use, based on a calculation of inverse yield and occupation time, as described in detail elsewhere [6].	m^2 per year
Greenhouse gas emissions	Emissions have been calculated based on the IPCC AR5 100-year factors.	kg CO_2-eq
Water scarcity	Use of scarce water resources, calculated using the AWARE method [12].	L-eq
Acidification potential	Calculations are based on either the CML2 baseline method (ingredients data) or the CML-IA baseline method (i.e. the 2016 update of the CML2 baseline method; used for the packaging data). However, calculations between these two datasets only differ for sulphuric acid and sulphur trioxide. These methods are comparable because the agricultural production of included ingredients does not produce sulphuric acid or sulphur trioxide emissions, and thus there is no meaningful difference between these indicators in this instance. More details are available online [13].	kg SO_2-eq
Eutrophication potential		kg PO_4^{3-} -eq
Plastic production	Warmerdam & Vickers presents the quantity of plastic per litre for each packaging type, which was multiplied by the quantity of packaging sold on the Australian market. This reflects the negative impacts of plastic production as described in the novel entities component of the planetary boundaries framework [14].	kg of plastic

3 Preparing data from Euromonitor

All Euromonitor data were downloaded on 03.08.23.

3.1 Packaging dataset

Data were available from Euromonitor regarding the types of packages used within broad, overarching product categories. However, our analysis excluded some products within these categories which were unable to be disaggregated. For example, we did not include 100% juices, which were a component of the 'Juice' packaging category. Thus, we determined the proportion of the types and sizes of packages within each category and applied these ratios to the relevant subcategories found in the sales dataset. This relies on the assumption that packaging is the same for e.g. 100% juices compared with juice drinks, which may not hold true. However, many of the product categorisations remained the same in both datasets (see Table S3).

Table S3: Alignment of Euromonitor packaging and sales data categories used in the analysis.

Packaging data category	Sales data category	Alignment of category definitions
Bottled water (UPFs and non-UPFs)	Carbonated bottled water (non-UPF)	Sales data are subcategories of the packaging data
	Still bottled water (non-UPF)	Sales data are subcategories of the packaging data
	Flavoured bottled water (UPF)	Sales data are subcategories of the packaging data
Asian Specialty Drinks (UPF)	Asian Specialty Drinks (UPF)	No difference between categories
Carbonates (UPF)	Carbonates (UPF)	No difference between categories
Energy drinks (UPF)	Energy drinks (UPF)	No difference between categories
RTD tea (UPF)	RTD tea (UPF)	No difference between categories
Sports drinks (UPF)	Sports drinks (UPF)	No difference between categories
Juice (UPFs and non-UPFs)	Juice drinks and reconstituted juice (UPF)	Sales data are subcategories of the packaging data

3.2 Ingredient and sales data

3.2.1 Determining inclusion of ingredients

Drawing upon standard LCA practices, we used a cut-off of 99% of the total beverage by weight, including packaging (15). Two exceptions to this rule occurred, resulting in additional exclusions. The rationale for these exclusions follows.

Tea

The values for tea in the Euromonitor database appeared to reflect a diluted tea flavouring based on an extract rather than whole tea leaves. We further investigated these products by sampling popular brands of iced tea sold in Australian supermarkets and found that their ingredient lists tended to list tea extract, rather than tea leaves, further validating this assumption. We subsequently excluded the tea extract/ flavouring due to a lack of data on their environmental impacts, and it being unlikely that the tea component of the extract (for which we had environmental impact data) would comprise >1% of the product category.

Plant extracts

Plant extracts were investigated using market research reports to understand common ingredients. Plant extracts and botanicals represent a wide range of plant-based flavourings. As there were limited available data on the types of botanicals used, as well as limited environmental impact data, the closest we could find was a combination of citrus fruit and tea to represent the range of foliage-based products and fruit-based ingredients that may be used to flavour these beverages. This decision was based on examples of botanicals described on a global sensory science company's website (16). However, as discussed above, only a small proportion of actual tea is likely used to flavour a product. Furthermore, citrus extracts are generally sourced from citrus peel which is a by-product of citrus production (17) and therefore has a negligible environmental impact. Thus, plant extracts were also excluded from the analysis.

All other ingredients which comprised >1% of the product by weight in any beverage category were included. Although the Euromonitor category of 'nectars' was excluded due to there being no available ingredient data.

3.2.2 Ingredient conversion factors

Some items required conversion factors to account for 1) the data being provided in weight instead of volume, 2) the product requiring reconstitution prior to consumption, or 3) due to differences in the agricultural commodity and final ingredients' weight. Conversion factors are outlined below.

Powder concentrates tonnes to million litres conversion

Only one product category required a weight-to-volume conversion. “Powdered concentrates” was comprised predominantly of powdered sports drinks; therefore, we assigned a conversion factor based on the weight-to-volume ratio of one scoop of “Powerade Mountain Blast Powder – 500g”. Weight (15.6g) was taken from the package and the volume (20mL) was measured using a package of Gatorade powder using electric kitchen scales, as this data was not readily available elsewhere.

Dilution factors

As our environmental assessment included the full product lifecycle (including consumption), dilution factors were applied to convert concentrates from their concentrated form (i.e. as sold) into the diluted form (i.e. as consumed) to determine the environmental intensities per litre. Dilution factors were sourced from the AUSNUT 2011-13 Food Recipe File [18] to determine the ratio of water to concentrate for a variety of beverages that are sold in a concentrated form.

- Liquid concentrate: 23% cordial (liquid concentrate), 77% water
- Powder concentrates: 5% sports drink, dry powder (powder concentrate), 95% water*

*note: the only powder concentrates recipes available from the AUSNUT 2011-13 Food Recipe File were labelled as ‘weaker than standard dilution’. However, the water-to-powder ratios in this recipe reflect the package instructions for both Gatorade and Powerade.

Proportion of commodity used throughout the beverage lifecycle

Factors were applied to fruit juice and maltodextrin to account for changes across the supply chain. For example, fruit juices were concentrated during ingredient processing, shipped while concentrated and subsequently reconstituted at the beverage manufacturing plant.

Maltodextrin is produced from wheat, corn and other commodities containing starch. Waste from the starch production process is limited [19]. However, the other parts of both the wheat germ and flour are processed into other items with value, which needs to be considered in the impact assessment. Both economic and mass allocation methods were considered for these purposes, however, mass allocation was deemed more appropriate due to fluctuations in the starch and gluten industries and difficulty accessing reliable economic information. Using mass allocation instead of economic allocations also aligns with decisions made in a previously conducted environmental impact assessment of wheat starch [20]. Unfortunately, the environmental impacts of maltodextrin produced from

Australian wheat were not available, thus we applied generic factors for processing wheat starches. Starch comprises 60-75% of the total wheat grain [21]. We allocated impacts based on the lower end of the starch values to account for parts of the starch that may be used to produce substances other than maltodextrin.

The final factors for these items follow.

- Fruit juice: ratio of 7kg of apples: 1kg of apple juice concentrate [22]: 7kg reconstituted apple juice [23]
- Maltodextrin: 60% allocation of impacts, by mass

We made no further assumptions regarding the proportions of ingredients used in our analysis.

4 Matching datasets

4.1 Packaging data

Packaging types and sizes listed in the Euromonitor database were matched with the closest possible size and packaging type available in the Warmerdam & Vickers report, scaled to the actual product size.

Results from the Warmerdam and Vickers data indicate that the weight of the PET bottles has substantial impacts on the study outcomes. Thus, we wanted to distinguish between regular weight PET bottles and lightweight PET bottles. The Euromonitor data does not distinguish between the PET bottle weights, thus we conducted additional research to determine the proportion of lightweight to regular PET bottles used within each product category. Warmerdam and Vickers found that the only category using lightweight PET bottles was 'Still Bottled Water' [5]. This was expected as thicker packaging is required to retain carbonation. Thus, we conducted an audit of the weight of PET packages used in the bottled water market in Australia. To ensure that lightweight PET packages were not used in other non-carbonated products, we also sampled the packaging of six still UPFs (juice drinks, cordials, iced teas and sports drinks; Table S4).

Our process for auditing the weight of PET bottles in the Australian market is as follows.

1. Leading bottled water brands (based on volume of sales in Australia) were identified via the Euromonitor database.
2. At least one bottle of still water of each from the top brands was purchased. Multiple bottles were included for brands that appeared to use packaging of different thicknesses.

3. Following the procedure outlined in Warmerdam and Vickers, at least one bottle from each of the leading brands was emptied, cleaned, dried and weighed using a Mettler Toledo AB204-S analytical balance scale (precise to 0.0001g, inspected annually).
4. Weights were compared with the weights (or calculated weights) of lightweight or regular weight PET bottles recorded by Warmerdam and Vickers [(5)].

Table S4 contains the findings. Results indicated that all but two bottled water products were packaged in lightweight PET plastic. The two products which did not qualify as lightweight also did not qualify for regular weight, and instead sat halfway between the two categories (classified as "semi-lightweight" in Table S4). Some of the lightweight PET water bottles were substantially lighter than the lightweight values (see Aldi 600mL & 1.5L, Coles 1.5L, Woolworths 1.5L in Table S4), thus the inclusion of semi-lightweight bottles within the lightweight category may balance some of the differences in weight within the category.

Despite specifically investigating UPFs with thinner packaging than similar products on the shelf, none of the UPFs were found to be packaged in lightweight PET bottles.

As a result, we assumed that 100% of the PET packaging used for bottled waters in our analysis were lightweight PET, while 100% of the PET packaging used for UPFs were regular weight PET.

Table S4: Values used to differentiate between lightweight and regular weight PET bottles

PET bottle category	Original weights from Warmerdam and Vickers	Calculated weights (g) of PET bottled water packaging, by pack size					
		500mL	600mL	750mL	1L	1.5L	2L
Lightweight value 1	22.65g per 1L			16.99		33.98	45.3
Lightweight value 2	15.30 per 600mL	12.75	15.30	19.13			
Regular weight value 1	43.44 per 1L	21.72	26.06	32.58		65.16	
Regular weight value 2	70.97 per 2L					53.23	
Regular weight value 3	32.00 per 250mL	64.00	76.80				

Table S5: Market share (Source: Euromonitor International), bottle size and weight (Source: own) and alignment with lightweight or regular weight PET bottles (Source: Warmerdam and Vickers) for each leading bottled water brand in Australia

Brand & flavour (where relevant)	Market share (% of 2022 retail volume)	Size of bottle	Weight	Bottle weight category
**Bottled water**				
Mount Franklin (Coca-Cola Co, The)	19.3	1.5L	33.00	Lightweight
		600mL	15.82	Lightweight
Frantelle (Asahi Group Holdings Ltd)	16.7	1L	27.12	Lightweight
Pump (Coca-Cola Co, The)	5.8	750mL	25.31	Semi-lightweight
Nu Pure (Nu-Pure Pty Ltd)	4.2	600mL	16.54	Lightweight
Cool Ridge (Asahi Group Holdings Ltd)	3.6	750mL	20.80	Lightweight

Nobles (Noble Beverages Pty Ltd)	3.5	2L	48.72	Lightweight
Evian (Danone, Groupe)	1.4	1L	36.15	Semi-lightweight
Private label – Aldi	37.3	1.5L	22.70	Lightweight
		600mL	10.40	Lightweight
Private label - Coles		1.5L	27.09	Lightweight
		1L	27.11	Lightweight
		600mL	12.81	Lightweight
Private label – Woolworths		1.5L	22.70	Lightweight
Others	8.1	-	-	-
**Sample of Still Bottled Water***				
Golden Circle Mango Nectar Fruit Drink	N/A	2L	70.13	Regular weight
Golden Circle Pine Mango Passion Cordial	N/A	2L	70.17	Regular weight
Lipton Peach Flavoured Iced Tea	N/A	500mL	26.18	Regular weight
Gatorade Active No Sugar Lemon Flavour	N/A	600mL	22.7	Regular weight
Gatorade Tropical	N/A	600mL	33.47	Regular weight
Powerade Ion 4	N/A	600mL	22.7	Regular weight

*products were sampled based on whether the bottled appeared to potentially be 'lightweight' (i.e. the PET packaging was weaker compared with similar products found in the supermarket).

4.2 Matching ingredient data

4.2.1 Main assumptions

We sourced the agricultural production environmental impact data from the Poore & Nemecek dataset [6]. These data were matched with the relevant ingredients in the Euromonitor ingredients dataset. Specific assumptions made throughout this process are listed below.

4.2.2 Assumptions regarding the commodity chosen for inclusion

Where Euromonitor was insufficiently disaggregated to determine the commodity used, data was sourced to inform decision-making. These assumptions were as follows:

- Sugars and Bulk Sweeteners (Euromonitor) were matched with cane sugar (Poore & Nemecek) as sugar cane is the main commodity used to produce sugars in Australia [24].
- Fruit or Fruit Juice (Euromonitor) was allocated 50% to oranges and 50% to apples (Poore & Nemecek)
  - No Euromonitor data were available for the most popular/ common reconstituted juice flavours in Australia. According to a survey of Australian consumers, the most popular juices among Australian consumers are orange juice (39%), followed by tropical (15%), apple (13%), breakfast juice (10%), pineapple (6%), or "other" (17%) [25]. Orange juice also comprises the largest proportion of sales (39% of sales) [26].
  - We further investigated the ingredients in tropical and breakfast juice. Golden Circle is the leading juice brand in Australia [Euromonitor International]. The main ingredients in Golden Circle Tropical Juice are: Pear or Apple juice (64%), Pineapple juice (28%), Orange juice (4.0%), Peach Puree (2.0%), Passionfruit (1.0%)], Paw Paw Puree (0.5%). In Golden Circle's Breakfast Juice the main ingredients are: apple (or pear) juice (53.9%), pineapple juice (29%), orange juice (15%), passionfruit (0.5%), banana puree (1.2%). All juice ingredients in both products are reconstituted. Based on these ingredients lists, and Australian consumer preferences for juice flavours, we estimated that 41.1% of the ingredients should be orange juice, 28% apple, 7% pineapple juice, 23% "other".
  - Pineapple juice was excluded because there was no environmental data in the Poore and Nemecek dataset for pineapples grown in Australia or South-East Asian countries, which is where the majority of pineapples consumed in Australia are grown [27]. Furthermore, this accounted for only a small proportion of the juice values, which were also only a small proportion of the overall product.
- Maltodextrin was matched with wheat
  - Maltodextrin can be produced from a range of starches including corn and wheat [28]. Australian wheat production substantially outweighs Australian corn production [27]. Thus, we assumed that the maltodextrin included in our analysis was produced from wheat.

4.2.3 Assumptions regarding the country of origin of ingredients

We determined the country of origin of the included ingredients using a combination of FAO STAT data (trade matrices and food balance sheets) and market research data. We assumed that 100% of each ingredient came from just one country, based on the largest supplier of each ingredient to Australia. A detailed breakdown of these decisions follows.

- Sugar: Australia
  - FAOSTAT data indicates that sugar is predominantly produced in Australia, from sugar cane, and very little is imported [(27)].
- Maltodextrin: Australia
  - No trade data for maltodextrin was found on FAOSTAT. According to market research, China is the global leader in maltodextrin production, where it is predominantly produced from corn [(29)]. However, according to FAOSTAT data, very little corn/ corn products are imported to Australia [(27)]. To avoid overestimating the environmental impacts of this product, we assumed that maltodextrin was produced in Australia, rather than China, which also informed the choice of wheat over corn.
- Apple Juice Concentrate: China; Orange Juice Concentrate: Brazil
  - Most Australian reconstituted juice is sourced from Brazil (orange juice) or China (apple juice) [(30)]. Thus, we assumed these were the countries of origin for the respective fruits.
  - We assumed that the fruits were processed in the country they were grown in.
  - These are reasonable assumptions given that the FAOSTAT trade data indicates that Brazil is a major producer of oranges and orange juice and that China is a major producer of both apples and apple juice concentrate [(31)].

4.2.4 Final decisions regarding the specific data point included in the analysis

As the Poore & Nemecek (2018) dataset draws on a wide variety of sources, there are multiple values for each commodity's environmental impacts. For Australian-produced items included in the dataset (sugar and maltodextrin from wheat), the values were relatively homogenous and thus an average value was taken. However, water scarcity values for Brazilian oranges and Chinese apples varied substantially.

After reviewing the background material for the data provided for Chinese apples, it became apparent that the values from one of the datasets were representative of the whole country [(32)], one was specific to a relatively low-production region [(33, 34)], while the third was unclear [(35)]. Thus, we chose the value that best represented the country-level impacts. This was also the median water scarcity footprint listed in Poore & Nemecek. We also utilised these values for the land and GHG emissions, however used the data with unknown representation for the acidification and eutrophication potentials due to missing data from other sources.

For Brazilian oranges, it was clear that some values were based on irrigated systems and others were rainfed. Thus, we applied a 39% weighting to the irrigated values and a 61% weighting to

non-irrigated values, which reflects the proportion of irrigated land used to produce oranges in Brazil in 2022 [36].

5 Sourcing additional data

5.1 Ingredient and beverage manufacturing

While data is available for subsequent supply chain stages in the Poore & Nemecek dataset, these values are not specific to the ingredients used in our analysis but rather reflect the whole-food commodity (e.g. apples instead of apple juice concentrate). Therefore, we sourced additional data to determine the environmental impacts of ingredient processing and transportation. Note that these data were only applied to calculate greenhouse gas emissions and therefore were excluded from other metrics (as per Figure 1 in the main text). Some data were available in the form of kg CO_2-eq, whereas other data were only available in the electricity used during production, which was subsequently multiplied by the greenhouse gas emissions based on the energy grid for the country of production. These data were downloaded from *Our World in Data*, which contains energy grid and emissions factors based on three primary sources (Ember's European Electricity Review 2022, Ember's Yearly Electricity data and BP's Statistical Review of World Energy 2022) [37]. The more recent data were preferred over older data, where possible, however, ultimately data throughout the analysis were sourced from varying years.

Data extensions to account for ingredient and beverage manufacturing were as follows:

- Direct values for the greenhouse gas emissions from ingredient manufacturing processes
    - Sugar: 0.03kg CO_2-eq/kg sugar
        - Used an average of Australian values from Poore & Nemecek
- Energy inputs used to calculate greenhouse gas emissions from ingredient manufacturing
    - Maltodextrin: 450.1 Wh electricity/kg starch
        - Based on values for processing wheat flour in Australia (from Poore & Nemecek) added to the values for producing wheat starch in Australia [38].
    - Fruit juice concentrate: 2.21 MJ electricity/ kg of reconstituted fruit juice
        - Based on values for apple juice concentrate production in Table 2 in Khanali, Kokei et al. [39].
- Energy required to manufacture beverages at the manufacturing/ bottling facility: 0.1 Wh/L of beverage [2].
- Energy grid emissions used to calculate GHGs, based on 2022 data
    - Australia: 503.17862 g CO_2/kWh
    - Brazil: 102.02703 g CO_2/kWh
    - China: 530.8478 g CO_2/kWh

5.2 Transportation of ingredients

Shipping distances were calculated using the Ports.com distance calculator [40]. We assumed items departed from and arrived at the largest container shipping ports in each respective country. These were the Port of Shanghai (China) [41], Porto de Santos (Brazil) [42] and Port of Melbourne (Australia) [43].

Transportation distances were multiplied by the transportation emissions per kilometre for the relevant vehicle type, as reported in Table 1 Weber & Matthews [44].

It was assumed all ingredients were transported without refrigeration. Fruit juice concentrate can be transported frozen or shelf stable. As data on the proportion of frozen versus shelf-stable fruit juice concentrate was unavailable, we assumed it was transported in the shelf-stable form to avoid any overestimation of emissions resulting from transport. Furthermore, we assigned no value to the storage as we assumed it was stored without refrigeration. In reality, a proportion of these products would be transported and stored frozen, so the final values are likely to underestimate these impacts. This may balance some other assumptions, such as the assumption that 100% of the fruit juice concentrates were sourced from overseas, even though a small proportion were likely locally produced.

6 Supplementary Tables and Figures

6.1 Supplementary Sales Figures

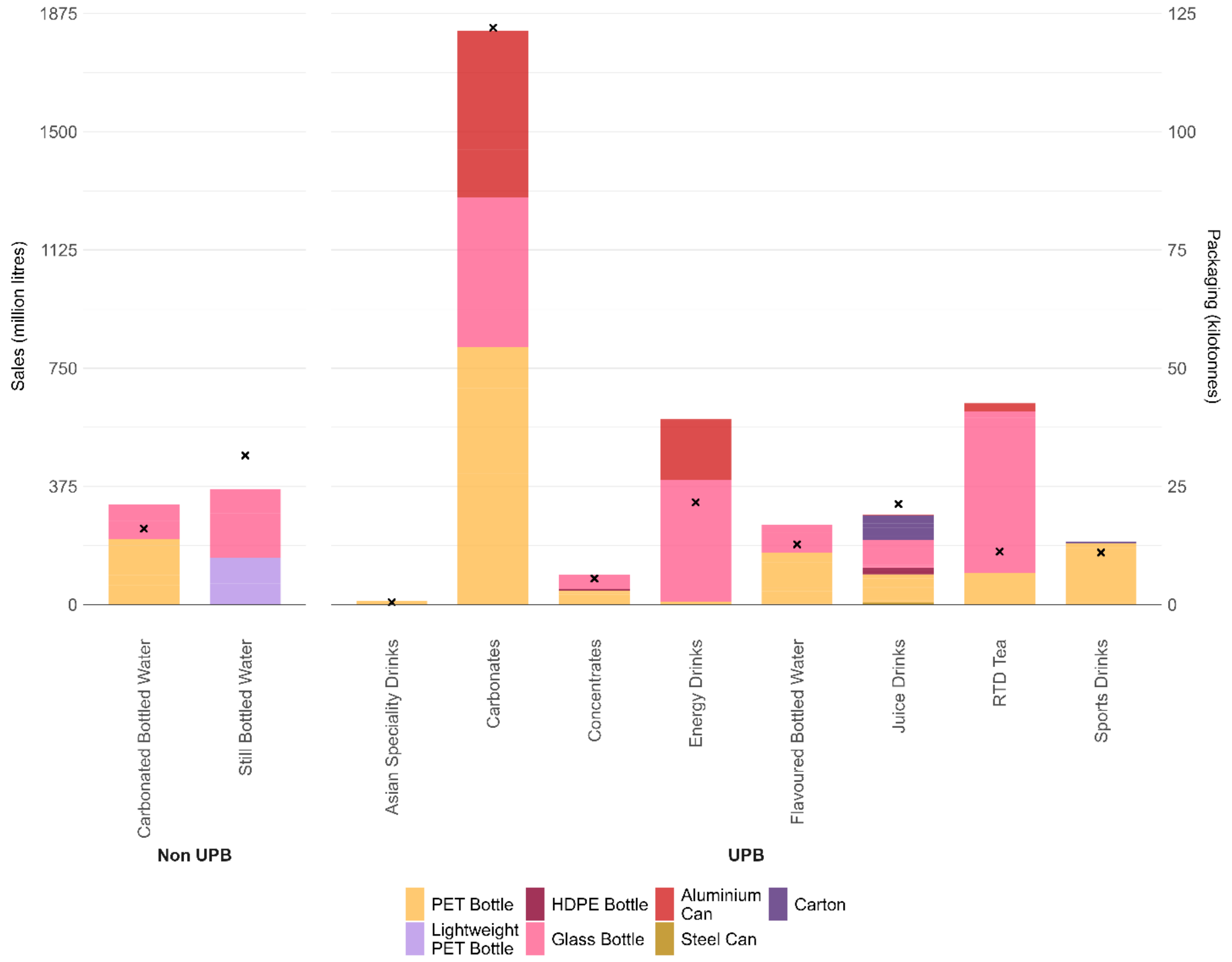

Supplementary Figure S1: Total volume of beverages project to be sold in Australia in 2027 and the associated quantity of consumer packaging.

Results are displayed according to beverage category. 'x' indicate the sales (million litres sold in 2027), bars refer to the packaging used (kilotonnes sold in 2027). UPB = ultra-processed beverage; RTD= ready-to-drink

6.2 Supplementary Environmental Impact Figures and Tables

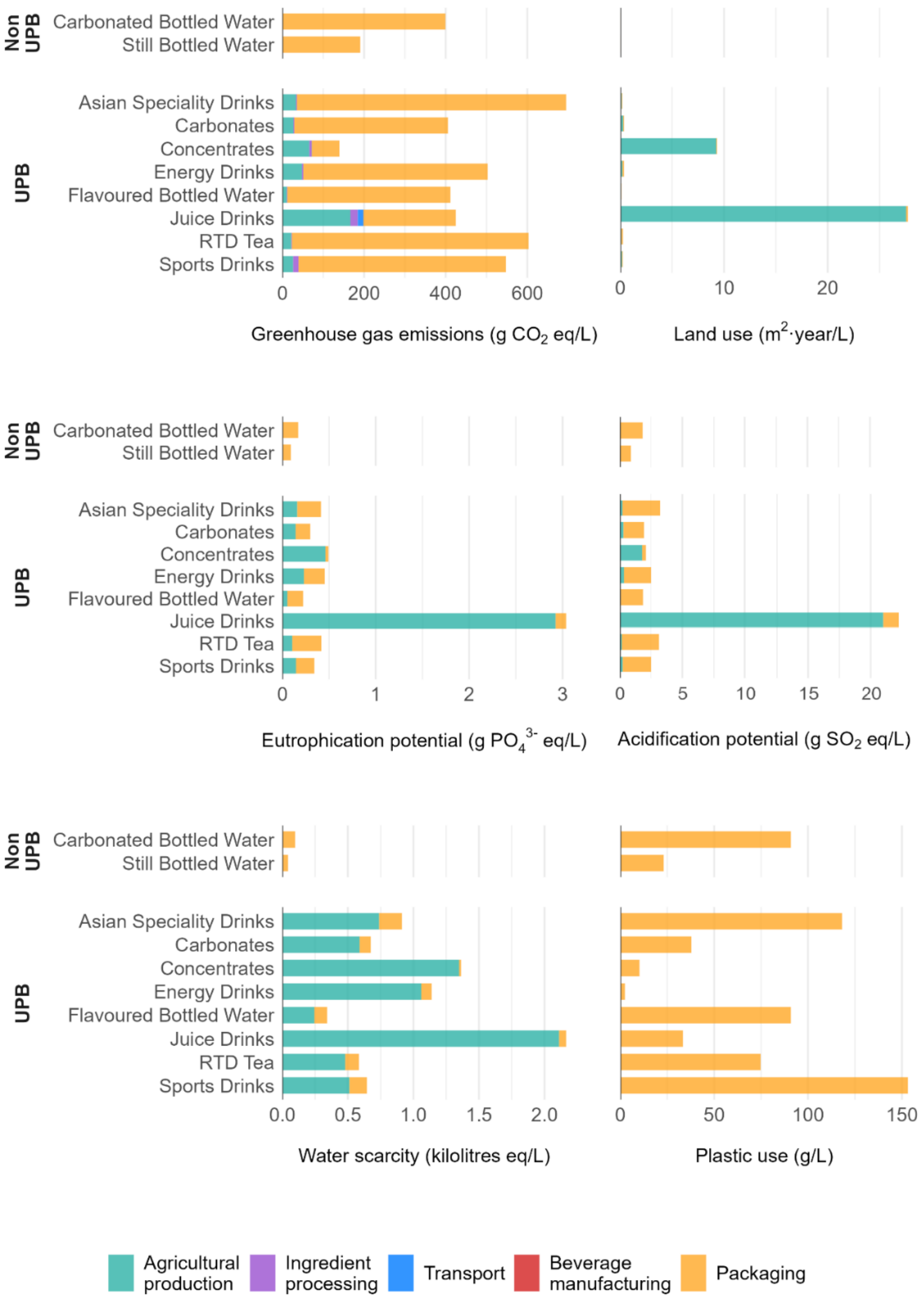

Supplementary Figure S2: Environmental intensities of beverages sold in Australian in 2022, per litre of content, grouped according to beverage categories.

Colours indicate the proportional impacts from each supply chain stage, illustrating that the majority of impacts stem from the agricultural production and packaging supply chain stages. Greenhouse gas emissions are expressed in carbon dioxide equivalents per litre of beverage, land use is expressed in metres-squared per litre of beverage, eutrophication potential is expressed in phosphate equivalents per litre of beverage, acidification potential is expressed in sulphur dioxide equivalents per litre of beverage, water scarcity is expressed in kilolitres equivalent per litre of beverage and plastic use, which exclusively applies to the consumer packaging (i.e. it does not account for plastic used throughout the packaging or ingredient supply chains), is measured in grams per litre of beverage. UPB = ultra-processed beverage; RTD= ready-to-drink.

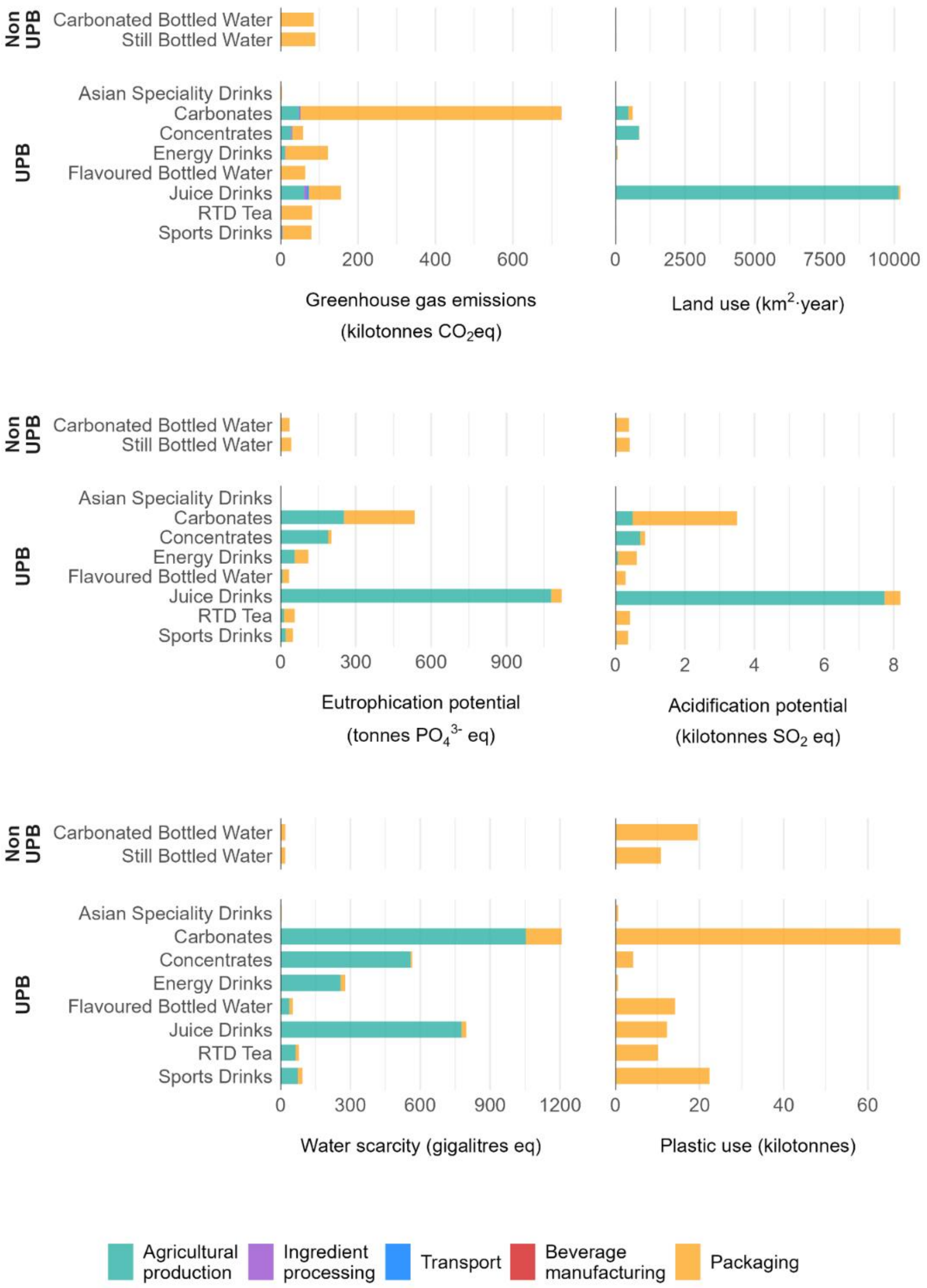

Supplementary Figure S3: Environmental impacts associated with the total volume of beverages sold in Australia in 2022, grouped according to beverage categories.

Colours indicate the proportional impacts from each supply chain stage, illustrating that the majority of impacts stem from the agricultural production and packaging supply chain stages. Greenhouse gas emissions are expressed in carbon dioxide equivalents, land use is expressed in metres-squared, eutrophication potential is expressed in phosphate equivalents, acidification potential is expressed in sulphur dioxide equivalents, water scarcity is expressed in kilolitres equivalent and plastic use, which exclusively applies to the consumer packaging (i.e. it does not account for plastic used throughout the packaging or ingredient supply chains), is measured in grams. UPB = ultra-processed beverage; RTD= ready-to-drink.

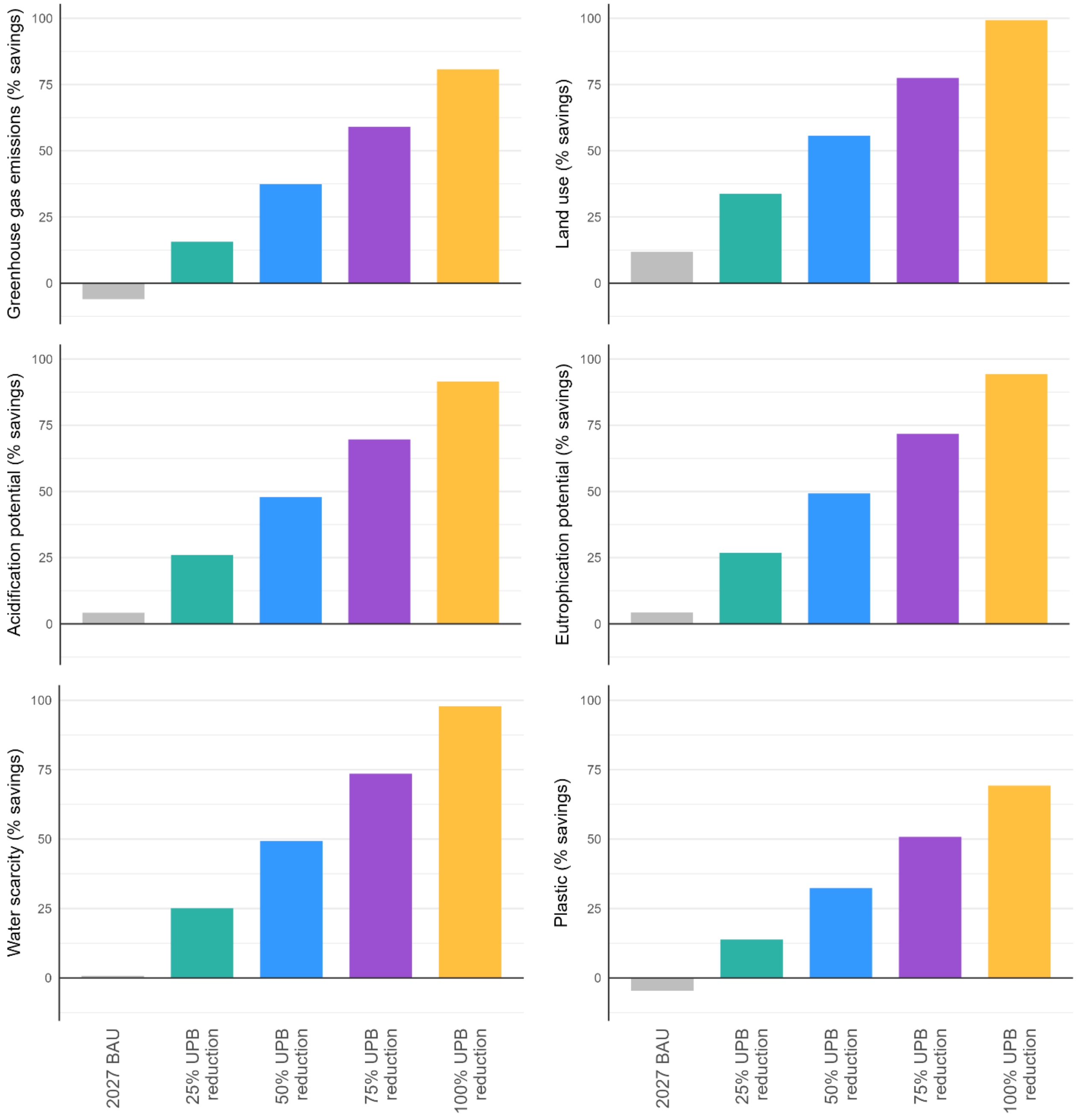

Supplementary Figure S4: Modelled environmental impacts of water-based beverages sold in Australia in 2027 under different UPB reduction scenarios.

All results are presented as percentage environmental savings compared with a 2022 baseline. Models assume that 50% of avoided UPBs are replaced with bottled waters and 50% with tap water. BAU= business as usual; UPB = ultra-processed beverage.

Supplementary Table S6: Environmental impacts associated with beverages sold in Australia in 2022.

Results are displayed by total impacts (discrete numerical values) and proportional contribution of each beverage category to the total environmental impacts of all included beverages (% total impacts). The blue-red gradient indicates the beverages which contributed to most environmental impact (dark red) through to least environmental impact (dark blue) per indicator. GHG= greenhouse gas emissions; AP = acidification potential; EP=eutrophication potential; WS= water scarcity; LU = land use; plastic = plastic use.

	**Analysis category**	**Total impacts**					
		**Total GHG (thousand tonnes CO_2 eq)**	**Total AP (tonnes SO2 eq)**	**Total EP (tonnes PO43- eq)**	**Total WS (gigalitres eq)**	**Total LU (m2.year)**	**Total plastic use (tonnes)**
**Non UPF**	Carbonated Bottled Water	86.2	391	36.6	21	14.7	19.6
	Still Bottled Water	89.9	413.6	42.9	20.3	33.9	10.9
**UPF**	Asian Speciality Drinks	3.8	17.7	2.3	5	1.1	0.6
	Carbonates	725.4	3498.8	534.7	1206.9	626.8	67.8
	Concentrates	58.4	852.5	203.3	565.8	858.4	4.2
	Energy Drinks	122.7	614.7	110.9	278	83.5	0.6
	Flavoured Bottled Water	64.2	293.2	34.7	53.4	17.5	14.2
	Juice Drinks	156.4	8194.2	1119	797.4	10220.9	12.3
	RTD Tea	81.7	425.4	56.6	79.1	33.7	10.1
	Sports Drinks	79.9	366.2	50	94.1	34.6	22.4

		% total impacts					
		Total GHG	Total AP	Total EP	Total WS	Total LU	Total plastic
Non UPF	Carbonated Bottled Water	6	3	2	1	0	12
	Still Bottled Water	6	3	2	1	0	7
	Total Non UPF	***12***	***5***	***4***	***1***	***0***	***19***
UPF	Asian Speciality Drinks	0	0	0	0	0	0
	Carbonates	49	23	24	39	5	42
	Concentrates	4	6	9	18	7	3
	Energy Drinks	8	4	5	9	1	0
	Flavoured Bottled Water	4	2	2	2	0	9
	Juice Drinks	11	54	51	26	86	8
	RTD Tea	6	3	3	3	0	6
	Sports Drinks	5	2	2	3	0	14
	Total UPF	***88***	***95***	***96***	***99***	***100***	***81***

Supplementary Table S7: Total impacts and percent savings of the environmental impacts of water-based beverages projected to be sold in Australia in 2027 under different policy-based scenarios and UPB reduction scenarios.

Scenario	Total impacts					
	GHG (kt CO_2 eq)	AP (tonnes SO_2 eq)	EP (tonnes PO_4^{3-} eq)	WS (gigalitres eq)	LU (km^2.y)	Plastic (kt)
2022 Baseline	1469	15067	2191	3121	11925	163
2027 Business as usual	1557	14436	2097	3096	10506	170
Reformulation only (2027)	1427	8292	961	952	2727	170
20% UPB reduction (2027)	1302	11806	1703	2490	8420	146
58% Recycled PET (2027)	1325	13485	2025	3047	10511	132
Mixed approach (2027)	1000	6079	733	733	2200	113
25% UPB reduction (2027)	1238	11149	1604	2339	7899	140
50% UPB reduction (2027)	920	7861	1112	1581	5291	110
75% UPB reduction (2027)	601	4574	619	824	2683	80
100% UPB reduction (2027)	282	1287	126	67	76	50

	Percent savings, compared with 2022 baseline (% savings)					
	**GHG**	**AP**	**EP**	**WS**	**LU**	**Plastic**
2022 Baseline	0	0	0	0	0	0
2027 Business as usual	-6	4	4	1	12	-5
Reformulation only (2027)	3	45	56	69	77	-5
20% UPB reduction (2027)	11	22	22	20	29	10
58% Recycled PET (2027)	10	10	8	2	12	19
Mixed approach (2027)	32	60	67	77	82	30
25% UPB reduction (2027)	16	26	27	25	34	14
50% UPB reduction (2027)	37	48	49	49	56	32
75% UPB reduction (2027)	59	70	72	74	77	51
100% UPB reduction (2027)	81	91	94	98	99	69

Data is displayed in both discrete numerical values and as percent savings compared with a 2022 baseline. Scenarios are described in the main text in Table 1. GHG= greenhouse gas emissions; AP = acidification potential; EP=eutrophication potential; WS= water scarcity; LU = land use; plastic = plastic use.

7 References

1. Anastasiou K, Baker P, Hendrie GA *et al.* (2023) Conceptualising the drivers of ultra-processed food production and consumption and their environmental impacts: A group model-building exercise. *Global Food Security* 37, 100688.

2. Amienyo D, Gujba H, Stichnothe H *et al.* (2013) Life cycle environmental impacts of carbonated soft drinks. *The International Journal of Life Cycle Assessment* 18, 77-92.

3. Fantin V, Scalbi S, Ottaviano G *et al.* (2014) A method for improving reliability and relevance of LCA reviews: The case of life-cycle greenhouse gas emissions of tap and bottled water. *Science of the Total Environment* 476-477, 228-241.

4. Tamburini E, Costa S, Summa D *et al.* (2021) Plastic (PET) vs bioplastic (PLA) or refillable aluminium bottles – What is the most sustainable choice for drinking water? A life-cycle (LCA) analysis. *Environmental Research* 196, N.PAG-N.PAG.

5. Warmerdam S & Vickers J (2021) *LCA of Beverage and Food Packaging in Australia and New Zealand*. Wellington, New Zealand: thinkstep Ltd, prepared for Tetra Pak Oceania.

6. Poore J & Nemecek T (2018) Reducing food's environmental impacts through producers and consumers. *Science*, 987.

7. Gleick PH & Cooley HS (2009) Energy implications of bottled water. *Environmental Research Letters* 4, 014009.

8. Muhammad N, Nafees M, Hussain R *et al.* (2018) Pollution and energy reduction strategy in soft drink industries. *Environmental science and pollution research international* 25, 28153-28159.

9. Pickin J, Wardle C, O'Farrell K *et al.* (2022) *National Waste Report 2022*. Canberra, Australia: The Department of Climate Change, Energy, the Environment and Water; Blue Environment Pty Ltd.

10. Grisales V, Tua C Rigamonti L (2021) Life cycle assessment of bottled mineral water for the hospitality industry in Northern Italy. *Packaging Technology & Science* 35, 301-314.

11. Hong C, Burney JA, Pongratz J *et al.* (2021) Global and regional drivers of land-use emissions in 1961–2017. *Nature* 589, 554-561.

12. Boulay A-M, Bare J, Benini L *et al.* (2018) The WULCA consensus characterization model for water scarcity footprints: assessing impacts of water consumption based on available water remaining (AWARE). *The International Journal of Life Cycle Assessment* 23, 368-378.

13. Universiteit Leiden. (2023) CML-IA Characterisation Factors. https://www.universiteitleiden.nl/en/research/research-output/science/cml-ia-characterisation-factors (accessed 24.05.2023

14. Persson L, Carney Almroth BM, Collins CD *et al.* (2022) Outside the Safe Operating Space of the Planetary Boundary for Novel Entities. *Environmental Science & Technology* 56, 1510-1521.

15. Environmental Product Declarations (2022) Product Category Rules: Soft Drinks, vol. PRODUCT CATEGORY CLASSIFICATION: UN CPC 24490, pp. 9-10.

16. Kerry Group. (2020) The Global State of Botanical Ingredients in Food and Botanical Beverages. https://www.kerry.com/insights/kerrydigest/2020/botanical-ingredients-in-food.html (accessed 21.04.2023

17. Kesbic O, Acar Ü, Mohammady E *et al.* (2022) The beneficial effects of citrus peel waste and its extract on fish performance and health status: A review. *Aquaculture Research* 53.

18. FSANZ (2013) AUSNUT 2011–13 food recipe file https://www.foodstandards.gov.au/science/monitoringnutrients/ausnut/ausnutdatafiles/Pages/foodrecipe.aspx

19. An V & Katrien B (2015) *Life Cycle Assessment study of starch products for the European starch industry association (Starch Europe): sector study*. Boeretang, Belgium: Vision on Technology (VITO); Starch Europe.

20. Deng Y, Achten WMJ, Van Acker K *et al.* (2013) Life cycle assessment of wheat gluten powder and derived packaging film. *Biofuels, Bioproducts and Biorefining* 7, 429-458.

21. Shevkani K, Singh N, Bajaj R *et al.* (2017) Wheat starch production, structure, functionality and applications—a review. *International Journal of Food Science & Technology* 52, 38-58.

22. Gale F, Huang S Gu Y (2010) *Investment in Processing Industry Turns Chinese Apples Into Juice Exports*. USDA.

23. Kerr Concentrates. (NP) Dilution Rate for Common Products [Kerr Concentrates., editor].

24. FSANZ (2019) Sugar. https://www.foodstandards.gov.au/consumer/nutrition/Pages/Sugar.aspx (accessed 26.04.2023

25. Canstar Blue. (2023) Best-Rated Fruit Juice. https://www.canstarblue.com.au/groceries/fruit-juice/ (accessed 17.05.2023

26. Goldstein Research. (2019) Australia Fruit Juice Market Size, Trends, Share, Industry Analysis, Demand & Growth Opportunity Assessment, Regional Outlook, & Forecast 2017-2025. https://www.goldsteinresearch.com/report/australia-fruit-juice-market (accessed 17.05.2023

27. FAOSTAT (2023) Food Balances (2010-). https://www.fao.org/faostat/en/#data/FBS (accessed 08.06.2023

28. Tiefenbacher KF (2017) Chapter Two - Technology of Main Ingredients—Water and Flours. In *Wafer and Waffle*, pp. 15-121 [KF Tiefenbacher, editor]: Academic Press.

29. Expert Market Research. (2022) Asia Pacific Maltodextrin Market Outlook. https://www.expertmarketresearch.com/reports/asia-pacific-maltodextrin-market (accessed 21.04.2023

30. Foulds T (2022) *Juice in Australia*. Euromonitor International.

31. FAOSTAT (2023) Detailed Trade Matrix. https://www.fao.org/faostat/en/#data/TM (accessed 08.06.2023

32. Xu X & Lan Y (2016) A comparative study on carbon footprints between plant- and animal-based foods in China. *Journal of Cleaner Production* 112, 2581-2592.

33. Yan M, Cheng K, Yue Q *et al.* (2016) Farm and product carbon footprints of China's fruit production—life cycle inventory of representative orchards of five major fruits. *Environmental Science and Pollution Research* 23.

34. USDA (2019) *Fresh Deciduous Fruit Annual*. no. CH2019-0145. Beijing, China: USDA; GAIN.

35. Nemecek T, Bengoa X, Lansche J *et al.* (2014) *World Food LCA Database Methodological Guidelines for the Life Cycle Inventory of Agricultural Products*.

36. USDA & GAIN (2022) *Citrus Semi-annual: Brazil*. no. BR2022-0041. Sao Paulo, Brazil: USDA, GAIN.

37. Ritchie H, Roser M Rosado P (2022) Energy. https://ourworldindata.org/electricity-mix (accessed 09.06.2023

38. Berkel Rv (2002) The Application of Life Cycle Assessment for Improving the Eco-Efficiency of Supply Chains *Muresk 75th Anniversary Conference*.

39. Khanali M, Kokei D, Aghbashlo M *et al.* (2020) Energy flow modeling and life cycle assessment of apple juice production: Recommendations for renewable energies implementation and climate change mitigation. *Journal of Cleaner Production* 246, 118997.

40. Ports.com. (2023) Sea route & distance. http://ports.com/sea-route/ (accessed 08.06.2023

41. Marine Insight. (2022) 10 Major Ports In China. https://www.marineinsight.com/know-more/10-major-ports-in-china/ (accessed 08.06.2023

42. Marine Insight. (2021) 10 Major Ports In Brazil. https://www.marineinsight.com/know-more/10-major-ports-in-brazil/ (accessed 08.06.2023

43. Port of Melbourne. (2023) Port of Melbourne – Facts and Figures. https://www.portofmelbourne.com/about-us/factsandfigures/ (accessed 08.06.2023

44. Weber CL & Matthews HS (2008) Food-Miles and the Relative Climate Impacts of Food Choices in the United States. *Environmental Science & Technology* 42, 3508-3513.
